# Risk factors from Framingham risk score for anthracyclines cardiotoxicity in breast cancer: A systematic review and meta-analysis

**DOI:** 10.3389/fcvm.2023.1101585

**Published:** 2023-01-19

**Authors:** Hao Jin, Jianfeng Xu, Zheng Sui, Lili Wang

**Affiliations:** ^1^Department of Cardiology, Second Affiliated Hospital of Dalian Medical University, Dalian, Liaoning, China; ^2^Department of Orthopedics, Affiliated Zhongshan Hospital of Dalian University, Dalian, China

**Keywords:** Framingham risk score, breast cancer, anthracycline-induced cardiotoxicity, cardiovascular risk factors, anthracycline

## Abstract

**Background:**

Framingham risk score (FRS) is an effective tool for evaluating the 10-year risk of cardiovascular diseases. However, the sensitivity of FRS for anthracycline-induced cardiotoxicity is unclear. This meta-analysis aims to evaluate the correlation between risk factors (hypertension, hyperlipidemia, diabetes, smoking, and obesity) in FRS and anthracycline-induced cardiotoxicity in breast cancer.

**Methods:**

We searched PubMed, EMBASE, and Cochrane Library for studies published from inception to January 2022 which reported cardiotoxicity due to anthracycline. Cardiotoxicity defined as any cardiac events were used as the primary endpoint. A total of 33 studies involving 55,708 breast cancer patients treated with anthracyclines were included in this meta-analysis.

**Results:**

At least one risk factor was identified at baseline for the 55,708 breast cancer patients treated with anthracycline. Hypertension [*I*^2^ = 45%, Fixed, RR (95% CI) = 1.40 (1.22, 1.60), *p* < 0.00001], hyperlipidemia [*I*^2^ = 0%, Fixed, RR (95% CI): 1.35 (1.12, 1.62), *p* = 0.002], diabetes [*I*^2^ = 0%, Fixed, RR (95% CI): 1.29 (1.05, 1.57), *p* = 0.01], and obesity [*I*^2^ = 0%, Fixed, RR (95% CI): 1.32 (1.05, 1.67), *p* = 0.02] were associated with increased risks of cardiac events. In addition, smoking was also associated with reduced left ventricular ejection fraction (LVEF) during anthracycline chemotherapy [*I*^2^ = 0%, Fixed, OR (95% CI): 1.91 (1.24, 2.95), *p* = 0.003] in studies that recorded only the odds ratio (OR).

**Conclusion:**

Hypertension, hyperlipidemia, diabetes, smoking, and obesity are associated with increased risks of anthracycline-induced cardiotoxicity. Therefore, corresponding measures should be used to manage cardiovascular risk factors in breast cancer during and after anthracycline treatment.

## 1. Background

Breast cancer is the most common cancer worldwide, which affects women most frequently in both developed and underdeveloped regions (1). Anthracyclines are widely used in the treatment of breast cancer (2), but anthracycline-induced cardiotoxicity is the main reason for their limited use in the clinical setting. Previous studies reported that the incidence of doxorubicin (DOX)-induced heart failure varies among individuals with different physical constitutions and is closely associated with the cumulative dose of anthracyclines and age of patients (3, 4).

The mechanisms of anthracycline-induced cardiotoxicity are complex and involve various processes of injury such as oxidative stress, inflammation (5), mitochondrial damage (6), endoplasmic reticulum stress (7), disrupted calcium homeostasis, cell apoptosis (8), fibrosis (5), and dysregulated autophagy (9, 10). In particular, reactive oxygen species (ROS) production plays an important role in anthracycline-induced cardiotoxicity. Several studies demonstrated that DOX can increase ROS production by cardiocytes *via* the nicotinamide adenine dinucleotide (NADH) dehydrogenase (complex I) pathway of the mitochondrial electron transport chain (11), increased mitochondrial iron level (12), down-regulation of sirtuin-3 (SIRT3), and decreased SOD2 production (13), ultimately resulting in cardiocyte apoptosis and increased autophagy (14).

Anthracycline-induced cardiotoxicity can be classified as acute cardiotoxicity (immediately after drug injection), early onset chronic progressive cardiotoxicity (during or within 1 year after treatment), and late-onset chronic progressive cardiotoxicity (at least 1 year after treatment). The most common manifestation of anthracycline-induced cardiotoxicity is left ventricular dysfunction (LVDF) and development of overt heart failure (15).

Echocardiography is the predominant tool for diagnosing cardiotoxicity and is required for evaluating left ventricular ejection fraction (LVEF) before, during and after treatment, especially in patients with cardiovascular risk factors (smoking, hypertension, diabetes, hyperlipidemia, and obesity) (16).

The Framingham risk score (FRS) is an effective tool for evaluating the risk of cardiovascular disease. This tool divides patients into the high-, medium-, and low-risk groups and estimates the 10-year risk of cardiovascular disease based on age and risk factors for cardiovascular disease. The risk factors included in the FRS are diabetes, hyperlipidemia, hypertension, smoking, and obesity (17). A guideline has suggested that the use of cardiovascular risk assessments to estimate the probability of future cardiovascular events may be beneficial for patients with risk factors.

In this study, we examined the effect of risk factors in the FRS on anthracycline-induced cardiotoxicity to determine the importance of cardiovascular risk factor assessment in anthracycline treatment of breast cancer patients.

## 2. Materials and methods

This study was conducted in accordance with the preferred reporting items for systematic reviews and meta-analyses (PRISMA) statement and was registered on PROSPERO (#CRD42022299098) (18).

### 2.1. Search strategy

Relevant studies in any language were searched in PubMed, Embase, and Cochrane Library from inception to January 2022 using the keywords [(anthracyclines or DOX or epirubicin) and (cardiac toxicity or cardiotoxicity or heart failure) and (breast or breast cancer)]. Case reports, reviews, guidelines, editorials, and letters were excluded.

### 2.2. Study selection

Two reviewers (JH and XJF) assessed the titles, abstracts, and full texts of the identified studies to determine whether the studies examined the associations between cardiovascular risk factors (hypertension, diabetes, hyperlipidemia, obesity, smoking, and obesity) and anthracycline-induced cardiotoxicity. We included cross-sectional and cohort studies as well as both population-based and hospital-based case-control studies in the systematic review. Eligible studies were identified if they met the following inclusion criteria: (1) adult participants ≥18 years of age and (2) all patients were treated with anthracycline. Studies were mainly excluded for the following reasons: (1) inappropriate study type, including reviews, editorials, and case report; (2) incomplete LVEF data; and (3) animal studies.

### 2.3. Data extraction

The extracted data included study environment, cohort description, incidence of cardiotoxicity, and cardiac event descriptions. Reduced LVEF was the primary endpoint. Abnormal electrocardiogram, duke activity status index (DASI) score, and congestive heart failure (CHF) were also included as endpoints in this meta-analysis.

### 2.4. Statistical analysis

The effect of five cardiovascular risk factors (hypertension, hyperlipidemia, diabetes, smoking, and obesity) on cardiotoxicity in breast cancer patients undergoing anthracycline treatment was separately pooled in this meta-analysis. Pooled results with an *I*^2^ < 50% were analyzed using the fixed effects model, while those with an *I*^2^ > 50% were analyzed by the random effects model. Subgroup analysis was also performed based on the clinical endpoint of each study. Sensitivity analysis was conducted on all studies with *I*^2^ > 50% to explore the source of heterogeneity and the effect of heterogeneity on the stability of the combined estimated value. Statistical analyses were performed on Review Manager 5.4 (The Nordic Cochrane Center, The Cochrane Collaboration) and STATA 16.0. In addition, publication bias was assessed using over 10 outcomes in the included studies by a funnel plot and Egger’s test.

## 3. Results

All statistical analysis results and literature quality evaluation (Supplementary Figure 34) are presented in two types of forest plots in the Supplementary Data, and only a summary of the results is shown in this manuscript (Tables 1, 2, 3).

**TABLE 1:** Risk ratio of cardiovascular risk factor for anthracycline-induced cardiotoxicity.

Cardiovascular factor	Endpoint	Study	Participants	*I* ^2^	*P*	Effect model	RR	*P*	Egger_*P*	TF
Hypertension	All cardiac event	23	4,748	45	0.01	Fixed effect	1.40 (1.22, 1.60)	<0.00001	0.713	Consistent
	LVDF	18	3,529	37	0.06	Fixed effect	1.27 (1.07, 1.52)	0.007	0.059	Consistent
	CHF	2	579	0	0.52	Fixed effect	1.11 (0.36, 3.42)	0.85	NA	NA
	Other cardiac event	3	640	79	0.008	Random effect	1.47 (0.93, 2.32)	0.1	NA	NA
Hyperlipemia	All cardiac event	12	3,176	0	0.58	Fixed effect	1.35 (1.12, 1.62)	0.002	0.522	Consistent
	LVDF	10	2,749	0	0.46	Fixed effect	1.24 (0.94, 1.63)	0.13	0.727	Consistent
	Other cardiac event	2	427	0	0.34	Fixed effect	1.48 (1.16, 1.90)	0.002	NA	NA
Diabetes	All cardiac event	20	4,534	0	0.83	Fixed effect	1.29 (1.05, 1.57)	0.01	0.483	Consistent
	LVDF	15	3,316	0	0.59	Fixed effect	1.14 (0.86, 1.51)	0.37	0.506	Consistent
	CHF	2	578	0	0.82	Fixed effect	2.31 (0.46,11.50)	0.31	NA	NA
	Other cardiac event	3	640	0	0.66	Fixed effect	1.51 (1.16, 1.96)	0.002	NA	NA
Smoke	All cardiac event	11	2,192	0	0.5	Fixed effect	1.04 (0.83, 1.30)	0.74	0.083	Consistent
	LVDF	8	1,510	0	0.43	Fixed effect	1.07 (0.81, 1.42)	0.63	NA	NA
	CHF	1	384	NA	NA	NA	0.50 (0.10, 2.36)	0.38	NA	NA
	Other cardiac event	2	298	1	0.31	Fixed effect	1.06 (0.73, 1.55)	0.77	NA	NA
Obesity	All cardiac event	9	3,598	0	0.61	Fixed effect	1.32 (1.05, 1.67)	0.02	NA	NA
	LVDF	6	1,719	0	0.92	Fixed effect	1.12 (0.86, 1.47)	0.4	NA	NA
	CHF	1	506	NA	NA	NA	1.42 (0.41, 4.97)	0.58	NA	NA
	Other cardiac event	2	1,373	0	0.97	Fixed effect	2.04 (1.26, 3.29)	0.004	NA	NA

LVDF, left ventricular dysfunction; CHF, congestive heart failure; RR, risk ratio.

**TABLE 2:** Odds ratio of cardiovascular risk factor for anthracycline-induced cardiotoxicity.

Cardiovascular factor	Endpoint	Population	Study	Effect model	*I*^2^ (%)	*P*	OR	*P*
Hypertension	LVDF	930	5	Fixed effect	0	0.52	2.38 (1.47, 3.86)	0.0004
Hyperlipidemia	LVDF	838	5	Fixed effect	0	0.46	1.35 (0.88, 2.07)	0.17
Diabetes	All cardiac event	4,126	6	Fixed effect	0	0.89	1.59 (1.15, 2.21)	0.005
	LVDF	838	5	Fixed effect	0	0.9	1.99 (1.05, 3.78)	0.04
Smoke	LVDF	838	5	Fixed effect	0	0.69	1.91 (1.24, 2.95)	0.003
Obesity	LVDF	767	3	Fixed effect	36	0.21	1.24 (0.67, 2.31)	0.5

LVDF, left ventricular dysfunction; CHF, congestive heart failure; OR, odds ratio.

**TABLE 3:** Hazards ratio of cardiovascular risk factor for anthracycline-induced cardiotoxicity.

Cardiovascular factor	Endpoint	Population	Study	Effect model	*I*^2^ (%)	*P*	HR	*P*
Hypertension	CHF	10,155	2	Fixed effect	0	0	1.45 (1.39, 1.52)	0
Diabetes	CHF	10,155	2	Fixed effect	0	0	1.74 (1.66, 1.83)	0

LVDF, left ventricular dysfunction; CHF, congestive heart failure; HR, hazards ratio.

### 3.1. Study selection and baseline characteristics

**FIGURE 1:**
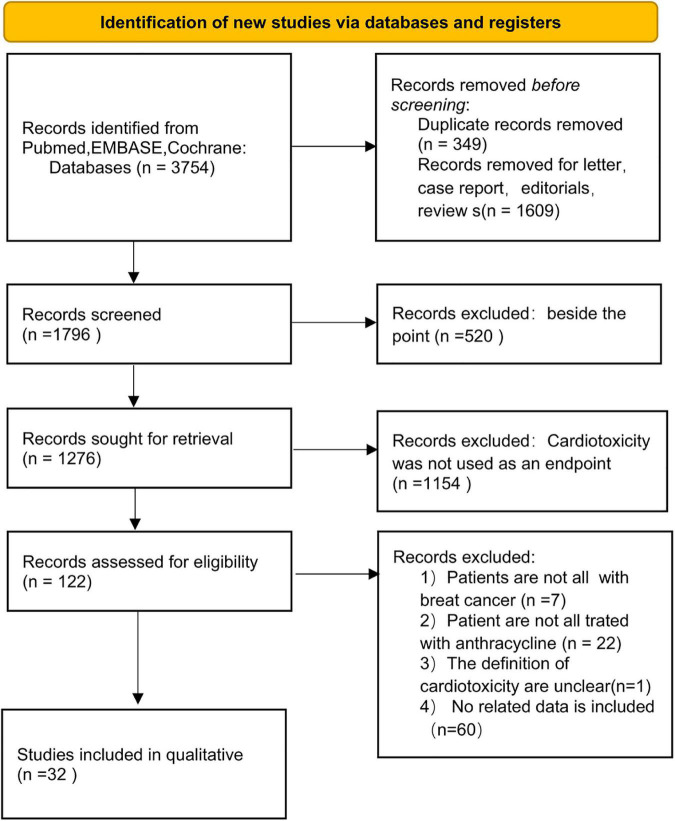
PRISMA flow diagram for systematic reviews.

The PRISMA flow diagram of study selection is shown in Figure 1. A total of 340 articles were identified during our preliminary search, and a final total of 32 eligible studies were included for meta-analysis. Detailed data including patient population, primary endpoint, and number of patients, were collected from the 32 studies (19–50). In addition, all patients in the included studies were breast cancer patients undergoing anthracycline treatment. Analysis of various risk factors showed that 30 studies [5 only recorded odds ratio (OR) and 2 recorded hazards ratio (HR)] reported hypertension, 18 studies (5 only recorded OR and 1 recorded) reported hyperlipidemia, 28 studies (6 recorded OR and 2 recorded HR) reported diabetes, 16 studies (5 included OR) reported smoking, and 12 studies (3 included OR) reported obesity (Table 4).

**TABLE 4:** Characteristics of the included studies.

References	Study design	No.	Anthracycline therapy	Type of breast cancer	Age	Baseline LVEF	Outcomes
Buzdar et al. (19)	Cross-sectional	534	Dox	Staged II to IV	NA	NA	CHF
Gennari et al. (20)	Cohort	105	Epi	Metastatic breast cancer	NA	NA	CHF
Pinder et al. (21)	Cross-sectional	43,329	Dox, Mito, Dauno, or Epi	Stage I to III	NA	NA	CHF
Perez et al. (22)	RCT	2,992	Dox	HER2+	NA	63.1 ± 6.3	CE
Sawaya et al. (23)	Cohort	43	Epi, Dox	HER2+	48.6 ± 10.1	65 ± 6	sLVEF (5, 55%), asLVEF (10, 55%)
Aitelhaj et al. (24)	Cohort	100	NA	HER2+	46.3 ± 10.0	70 ± 10.0	LVEF (10, 50%)
Chung et al. (25)	Cohort	174	Dox	Staged I to IV	52 ± 10	63.9 ± 5.1	LVEF < 50% or HF
Vivenza et al. (26)	Cross-sectional	48	Epi	Early stage	56.2 ± 10.1	62 ± 6	sLVEF (5, 55%)
Caram et al. (27)	Cross-sectional	165	Dox	Staged I to III	55.9 ± 9.8	55.9 ± 9.8	LVEF < 55%
Serrano et al. (28)	Cross-sectional	85	Epi, Dox	NA	49.7 ± 9.0	67.3 ± 5.7	Diastolic dysfunction
Mina et al. (29)	Cohort	220	Dox	Stage I to III	NA	NA	LVEF < 50%
Kotwinski et al. (30)	Cohort	165	Epi, Dox	Early stage	48.3 ± 8.9	NA	LVEF↓ ≥ 10% or heart failure
Matos et al. (31)	Cohort	92	Epi, Dox	HER2+	53.6 ± 9.0	66.3 ± 5.8	LVEF↓ ≥ 10% or heart failure
Reinbolt et al. (32)	Case control	162	NA	Staged I to III	50.7 ± 10.2	NA	LVEF↓ ≥ 15% or LVEF < 50%
Ganz et al. (33)	Cohort	342	Dox	Node + breast cancer	NA	NA	Dasi score
He (34)	Case control	213	Pir, Epi	NA	47.8 ± 9.07	NA	DEC abnormality
Kim et al. (35)	Cross-sectional	175	Dox	HER2+	52.4 ± 8.9	59.76 ± 3.48	sLVEF (10, 45%)
Pearson et al. (36)	Cohort	411	Dox	Staged I to IV	43.1 ± 9.7	NA	LVEF < 50% or CHF
Stachowiak et al. (37)	Cohort	75	Epi, Dox	NA	NA	62.1 ± 5	sLVEF (5, 55%) asLVEF (10, 55%)
Wu et al. (38)	Cohort	746	Dox	NA	48.7 ± 7.6	NA	CHF
El-Sherbeny et al. (39)	Cohort	61	Dox	HER2+	NA	62.7 ± 2.4	sLVEF (10, 55%)
Li et al. (40)	Cohort	427	Epi	Staged I to III	45.3 ± 6.0	67.0 ± 1.2	LVEF↓ ≥ 10% and LVEF < 53% or CE
Santos et al. (41)	Cross-sectional	231	Dox	NA	NA	NA	LVEF (10, 55%)
Fogarassy et al. (42)	Cohort	3,288	Dox	NA	NA	NA	LVEF↓ ≥ 15%
Rüger et al. (43)	Cross-sectional	853	Epi, Dox	Early stage	62.1 ± 5	65.5 ± 5.9	ICD-10 I50 (heart failure)
Tan et al. (44)	Case control	91	Epi	HER2+	50.4 ± 7.4	67.1 ± 1.4	LVEF↓ ≥ 10%
Todorova et al. (45)	Cohort	51	Dox	Early stage	52.2 ± 11.5	64.5 ± 7.0	LVEF↓ ≥ 10% and LVEF < 53% or CE
Vaitiekus et al. (46)	Case control	73	Dox	Staged I to III	55.4 ± 9.8	NA	LVEF↓ ≥ 10%
Cho et al. (47)	Cohort	613	Dox	Staged I to IV	54.1 ± 9.7	66.0 ± 5.8	LVEF↓ ≥ 10%
Egashira et al. (48)	Case control	64	Epi, Dox	NA	52.6 ± 10.6	59.3 ± 11.0	LVEF (10, 53%)
Feng et al. (49)	Cohort	72	Epi	HER2+	52.3 ± 7.6	68.6 ± 4.3	LVEF (10, 53%)
Houbois et al. (50)	Cohort	125	NA	HER2+	50.9 ± 9.0	62.9 ± 4.0	LVEF (10, 55%)

RCT, randomized controlled trial; CHF, congestive heart failure; HF, heart failure; LVEF, left ventricular ejection fraction; LEVF↓, decreasing of LVEF from baseline; Dox, doxorubicin; Epi, epirubicin; Mito, mitoxantrone; Dauno, daunorubicin; Pir, pirarubicin; CE, cardiac event; s, symptomatic; as, asymptomatic.

### 3.2. Incidence of cardiotoxicity

Cardiotoxicity was observed in 11,516 of the 55,492 patients included in this meta-analysis (Rate: 0.20; 95% CI: 0.15–0.24) with high heterogeneity (*I*^2^ = 99%). The result remained unchanged after sensitivity analysis (Supplementary Figure 1).

#### 3.2.1. Hypertension

We analyzed 23 studies involving 4,748 patients (Supplementary Figure 2) and confirmed that hypertension was a risk factor for cardiotoxicity [*I*^2^ = 45%, Fixed, RR (95% CI): 1.40 (1.22, 1.60), *p* < 0.00001].

Subgroup analysis using hypertension as the clinical endpoint showed that 18 studies involving 3,529 patients used LVDF as the endpoint [*I*^2^ = 37%, Fixed, RR (95% CI): 1.27 (1.07, 1.52), *p* = 0.007] (Supplementary Figure 3) and 2 studies involving 579 patients [*I*^2^ = 0%, Fixed, RR (95% CI): 1.11 (0.36, 3.42), *p* = 0.85] used CHF as the endpoint (Supplementary Figure 4).

There were three studies involving 640 patients [*I*^2^ = 79%, Random, RR (95% CI): 1.47 (0.93, 2.32), *p* = 0.1] that used other cardiac events as the clinical endpoints (Supplementary Figure 5). Sensitive analysis revealed that the Ganz et al. (33) study was the main source of heterogeneity and was hence removed [*I*^2^ = 0%, Fixed, RR (95% CI): 1.17 (0.87, 1.56), *p* = 0.3]. The reason for heterogeneity may be attributed to the large difference in clinical endpoint between this study and other studies (described as unable to reduce heterogeneity in the sensitivity analysis).

There were six studies involving 526 HER2+ patients [*I*^2^ = 0%, Fixed, RR (95% CI): 1.14 (0.77, 1.68), *p* = 0.52; Supplementary Figure 34].

There were 12 studies involving 2,720 patients [*I*^2^ = 46%, Fixed, RR (95% CI): 1.77 (1.47, 2.13), *p* < 0.00001] that used DOX (Supplementary Figure 35).

Of the studies that recorded only OR and HR, five studies [pooled: *I*^2^ = 0%, Fixed, OR (95% CI): 2.38 (1.47, 3.86), *p* = 0.0004; Supplementary Figure 6] and two studies [pooled: *I*^2^ = 0%, Fixed, HR (95% CI): 1.45 (1.39, 1.52), *p* = 0; Supplementary Figure 7] indicated that hypertension was a risk factor for cardiotoxicity, respectively.

#### 3.2.2. Hyperlipidemia

Analysis of 12 studies involving 3,176 patients (Supplementary Figure 8) showed that hyperlipidemia was a risk factor for cardiotoxicity [*I*^2^ = 0%, Fixed, RR (95% CI): 1.35 (1.12, 1.62), *p* = 0.002]. LVDF was the endpoint in 10 studies (Supplementary Figure 9) involving 2,749 subjects [*I*^2^ = 0%, Fixed, RR (95% CI): 1.24 (0.94, 1.63), *p* = 0.13], and other cardiac events were the endpoints in 2 studies (Supplementary Figure 10) involving 427 subjects [*I*^2^ = 0%, Fixed, RR (95% CI): 1.48 (1.16, 1.90), *p* = 0.002].

There were three studies involving 229 HER2+ patients [*I*^2^ = 45%, Fixed, RR (95% CI): 1.44 (0.89, 2.35), *p* = 0.14; Supplementary Figure 36].

There were six studies involving 1,864 patients [*I*^2^ = 0%, Fixed, RR (95% CI): 1.37 (1.09, 1.72), *p* = 0.007] that used DOX (Supplementary Figure 37).

Of the studies that recorded OR (Supplementary Figure 11), included five studies [pooled: *I*^2^ = 0%, Fixed, OR (95% CI): 1.35 (0.88, 2.07), *p* = 0.17].

#### 3.2.3. Diabetes

Analysis of 20 studies (Supplementary Figure 12) involving 4,534 patients indicated that diabetes was a risk factor for cardiotoxicity [*I*^2^ = 0%, Fixed, RR (95% CI): 1.29 (1.05, 1.57), *p* = 0.01].

Subgroup analysis using diabetes as the clinical endpoint showed that 15 studies (Supplementary Figure 13) involving 3,316 subjects [*I*^2^ = 0%, Fixed, RR (95% CI): 1.14 (0.86, 1.51), *p* = 0.37] used LVDF as the endpoint, 2 studies (Supplementary Figure 14) involving 578 subjects [*I*^2^ = 0%, Fixed, RR (95% CI): 2.31 (0.46, 11.50), *p* = 0.31] used CHF as the endpoint, and 3 studies (Supplementary Figure 15) involving 640 patients [*I*^2^ = 0%, Fixed, RR (95% CI): 1.51 (1.16, 1.96), *p* = 0.002] used other cardiac events (abnormal ECG, diastolic dysfunction, and all cardiac event) as the endpoints. The main source of heterogeneity was the difference in clinical endpoints.

There were four studies involving 329 HER2+ patients [*I*^2^ = 0%, Fixed, RR (95% CI): 1.39 (0.81, 2.39), *p* = 0.23; Supplementary Figure 38].

There were 10 studies involving 2,729 patients [*I*^2^ = 0%, Fixed, RR (95% CI): 1.22 (0.93, 1.61), *p* = 0.15] that used DOX (Supplementary Figure 39).

Of the studies that recorded only OR (Supplementary Figure 16), six confirmed that diabetes was a risk factor for cardiotoxicity [pooled OR: *I*^2^ = 0%, Fixed, OR (95% CI): 1.59 (1.15, 2.21), *p* = 0.005]. Of these six studies, five used LVDF as the endpoint (Supplementary Figure 17) and confirmed that diabetes was a risk factor for cardiotoxicity [pooled OR: *I*^2^ = 0%, Fixed, OR (95% CI): 1.99 (1.05, 3.78), *p* = 0.04]. Among the studies that recorded HR, two (Supplementary Figure 18) indicated that diabetes was a risk factor for cardiotoxicity [pooled HR: *I*^2^ = 0 %, Fixed, HR (95% CI): 1.74 (1.66, 1.83), *p* = 0].

#### 3.2.4. Smoking

Analysis of 11 studies (Supplementary Figure 19) involving 2,192 patients [*I*^2^ = 0%, Fixed, RR (95% CI): 1.04 (0.83, 1.30), *p* = 0.74].

Subgroup analysis using smoking as the clinical endpoint showed that eight studies (Supplementary Figure 20) involving 1,510 subjects [*I*^2^ = 0%, Fixed, RR (95% CI): 1.07 (0.81, 1.42), *p* = 0.63] used LVDF as the endpoint, and two studies (Supplementary Figure 21) involving 298 subjects [*I*^2^ = 1%, Fixed, RR (95% CI): 1.06 (0.73, 1.55), *p* = 0.77] used other cardiac events (abnormal ECG, diastolic dysfunction, and all cardiac event) as the endpoints.

There were three studies involving 229 HER2+ patients [*I*^2^ = 0%, Fixed, RR (95% CI): 1.39 (0.86, 2.25), *p* = 0.18; Supplementary Figure 40].

There were five studies involving 1,490 patients [*I*^2^ = 39%, Fixed, RR (95% CI): 0.96 (0.61, 1.51), *p* = 0.85] that used DOX (Supplementary Figure 41).

Five studies (Supplementary Figure 22) with OR data indicated that smoking was a risk factor for cardiotoxicity [pooled OR: *I*^2^ = 0%, Fixed, OR (95% CI): 1.91 (1.24, 2.95), *p* = 0.003].

#### 3.2.5. Obesity

Analysis of nine studies (Supplementary Figure 23) involving 3,598 patients demonstrated that obesity is a risk factor for cardiotoxicity [*I*^2^ = 0%, Fixed, RR (95% CI): 1.32 (1.05, 1.67), *p* = 0.02].

Subgroup analysis using obesity as the clinical endpoint showed that six studies (Supplementary Figure 24) involving 1,719 subjects [*I*^2^ = 0%, Fixed, RR (95% CI): 1.12 (0.86, 1.47), *p* = 0.4] used LVDF as the endpoint, and two studies (Supplementary Figure 25) involving 1,373 subjects [*I*^2^ = 0%, Fixed, RR (95% CI): 2.04 (1.26, 3.29), *p* = 0.004] used other cardiac events as the endpoints.

There were five studies involving 2,634 patients [*I*^2^ = 0%, Fixed, RR (95% CI):1.38 (0.92, 2.07), *p* = 0.12] that used DOX (Supplementary Figure 42).

Three studies with OR data (Supplementary Figure 26) [pooled OR: *I*^2^ = 36%, Fixed, OR (95% CI): 1.24 (0.67, 2.31), *p* = 0.5].

#### 3.2.6. Publication bias

Funnel plots were generated for cardiovascular risk factor with >10 included studies, including hypertension (23 studies), hyperlipidemia (12 studies), diabetes (20 studies), and smoking (11 studies), and all of the plots were symmetrical (Supplementary Figures 27–30).

Among the studies that used LVDF as endpoint, funnel plots for hypertension (18 studies), hyperlipidemia (10 studies), and diabetes (15 studies) were also symmetrical (Supplementary Figures 31–33).

Among the studies that used DOX, funnel plots for hypertension (12 studies) and diabetes (10 studies) were symmetrical (Supplementary Figures 43, 44).

## 4. Discussion

This meta-analysis examined the correlation between five cardiovascular risk factors in the FRS and anthracycline-induced cardiotoxicity in breast cancer patients, and fond that hypertension, hyperlipidemia, diabetes, smoking, and obesity were significantly associated with cardiotoxicity. Most included studies used decreased LVEF as the clinical endpoint. Subgroup analysis of studies with large heterogeneity in hypertension and smoking showed that abnormal ECG, diastolic dysfunction, and DASI score (indicators for cardiotoxicity) were the main sources of heterogeneity. Therefore, these clinical endpoints can be further examined in subsequent studies to improve the detection rate of cardiotoxicity. The pooled incidence of cardiac event (mean: 0.20; 95% CI: 0.15–0.24) in this study was similar to a recent meta-analysis (51) but with significant heterogeneity, which was possibly related to the differences in chemotherapy regimen, follow-up time and types of breast cancer.

Our meta-analysis revealed that cardiotoxicity was more easily induced by anthracyclines in breast cancer patients with hypertension, and this phenomenon can be caused by multiple factors. The synergistic effects of excess ROS production, DOX, and hypertension on the renin-angiotensin system (RAS) may be an important potential mechanism. Several studies demonstrated that DOX-induced cardiotoxicity was more severe in animals with hypertension than in normal animals (52, 53). Furthermore, a recent study showed that DOX and angiotensin-II (ANGII) exert synergistic effect in adolescent mice, and exposure to DOX can increase ANGII-induced cardiac remodeling (54). The putative mechanism underpinning this observation may be the changes in RAS induced by hypertension and DOX, which synergistically exacerbate cardiac remodeling (55). Nicotinamide adenine dinucleotide phosphate (NADPH) produced during ventricular hypertrophy in hypertension patients is the main source of ROS (56, 57). Prior study showed that ROS production is increased in the heart tissues of mice with spontaneous hypertension (58), which suggests that the increase in ROS during anthracycline treatment may result in cardiac injury in hypertension patients.

This study also demonstrated that cardiotoxicity is more easily induced by anthracyclines in patients with hyperlipidemia, which may be attributed to oxidative overload caused by hyperlipidemia. Jia et al. (59) reported that palmitate exposure in H9C2 cardiomyocytes led to increased cardiomyocyte apoptosis as a result of oxidative stress caused by ROS produced during lipid peroxidation. Zbinden et al. (60) showed that rats on high-fat diet (HFD) had more severe cardiotoxicity following intraperitoneal DOX injection than rats on low-FD, and this may be attributed to oxidative stress caused by ROS generation during lipid peroxidation and the production of excess ceramide (61).

We also identified diabetes as a risk factor for anthracycline-induced cardiotoxicity in breast cancer patients. However, inflammatory damages to the heart caused by diabetes may also contribute to the development of cardiotoxicity. It was shown that diabetic rats had decreased plasma and renal clearance of DOX and increased cardiotoxicity than normal rats (62). Similarly, another study demonstrated that diabetic mice had a higher risk of cardiotoxicity after DOX injection than normal mice (63). Given that diabetic patients already have up-regulated inflammation-associated protein expression in the heart, increased oxidative stress (59) can synergize with anthracyclines to exacerbate cardiac injury. However, since direct evidence linking these factors and anthracycline-induced cardiotoxicity is lacking, further investigation is warranted.

We showed that smoking is a risk factor for anthracycline-induced cardiotoxicity in breast cancer patients, possibly due to the compounds generated in smoke exposure. A previous study demonstrated that smoking plays an important role in anthracycline-induced cardiotoxicity. The authors found that exposure of anthracycline-treated cardiomyocytes to cigarette smoke led to increased concentrations of two compounds related to cardiac atrophy (64). Likewise, our meta-analysis revealed that studies that examined smoking and only included OR confirmed an association between smoking and anthracycline-induced cardiotoxicity.

Finally, our study demonstrated that obesity also increased the risk of anthracycline-induced cardiotoxicity in breast cancer patients, and patients with smaller body mass index (BMI) had lower risk of anthracycline-induced cardiotoxicity. A previous clinical study reported that obesity was associated with adverse outcome in node-positive breast cancer patients (65). In addition, rats with HFD-induced obesity were more susceptible to adriamycin-induced cardiotoxicity (66). The possible mechanism by which obesity increases cardiotoxicity is the down-regulation of adiponectin and omentin in obese patients (67–69), and calorie restriction and exercise have been shown to effectively decrease cardiac injury in these patients (70, 71).

Compared with previous studies, our meta-analysis included more studies and evaluated five cardiovascular risk factors. However, there are still several limitations. First, we could not examine the relationship between gender and anthracyclines due to the small number of male breast cancer patients. Second, despite the definition of cardiotoxicity in relevant guidelines, cardiotoxicity was still not defined consistently in some studies. Third, the chemotherapy regimen in most studies involved other chemotherapeutic agents, which impeded us from evaluating the correlation between a single anthracycline drug and cardiotoxicity in breast cancer patients. Last, since the risk factors analyzed in this study were dichotomous and not continuous variables, the relationship between the severity of risk factors and cardiotoxicity could not be analyzed.

In sum, our findings suggest that cardiovascular risk factors in breast cancer patients should be adequately assessed before anthracycline chemotherapy to evaluate the risk of cardiotoxicity in these patients. In addition, the cardiovascular system of breast cancer patients should also be closely monitored during anthracycline treatment.

## 5. Conclusion

Five cardiovascular risk factors from the FRS are highly associated with anthracycline-induced cardiotoxicity. Therefore, active management of the primary disease and maintenance of a good lifestyle can lower the risk of cardiotoxicity.

## Data availability statement

The original contributions presented in this study are included in this article/Supplementary material, further inquiries can be directed to the corresponding authors.

## Ethics statement

Ethical review and approval was not required for this study in accordance with the local legislation and institutional requirements.

## Author contributions

HJ: conceptualization, methodology, software, writing – original draft, data curation, and visualization. JX: investigation, writing – original draft, and writing – reviewing and editing. ZS: methodology, software, and writing – original draft. LW: conceptualization, supervision, project administration, and funding acquisition. All authors contributed to the study conception, design, read, and approved the final manuscript.
