# Supplementary material for: Risk factors from Framingham risk score for anthracyclines cardiotoxicity in breast cancer: A systematic review and meta-analysis
Source: Front Cardiovasc Med. 2023 Jan 19;10:1101585. doi: 10.3389/fcvm.2023.1101585 (PMC9892715; doi:10.3389/fcvm.2023.1101585)
Supplement: Supplementary file 1 [file Data_Sheet_1.docx]

**Supplementary material**

Contents

[Supplementary Figures 2](#_Toc123075782)

[Literature search strategy 24](#_Toc123075783)

[PUBMED 24](#_Toc123075784)

[EMBASE 25](#_Toc123075785)

[COCHRANE 28](#_Toc123075786)

[Literature quality evaluation 30](#_Toc123075787)

[case control study 30](#_Toc123075788)

[cross-sectional study 31](#_Toc123075789)

[randomized controlled trial 32](#_Toc123075790)

[cohort studies 33](#_Toc123075791)

[Literature inclusion and exclusion 35](#_Toc123075792)

[Literature inclusion criteria 35](#_Toc123075793)

[Literature exclusion 35](#_Toc123075794)

## Supplementary Figures


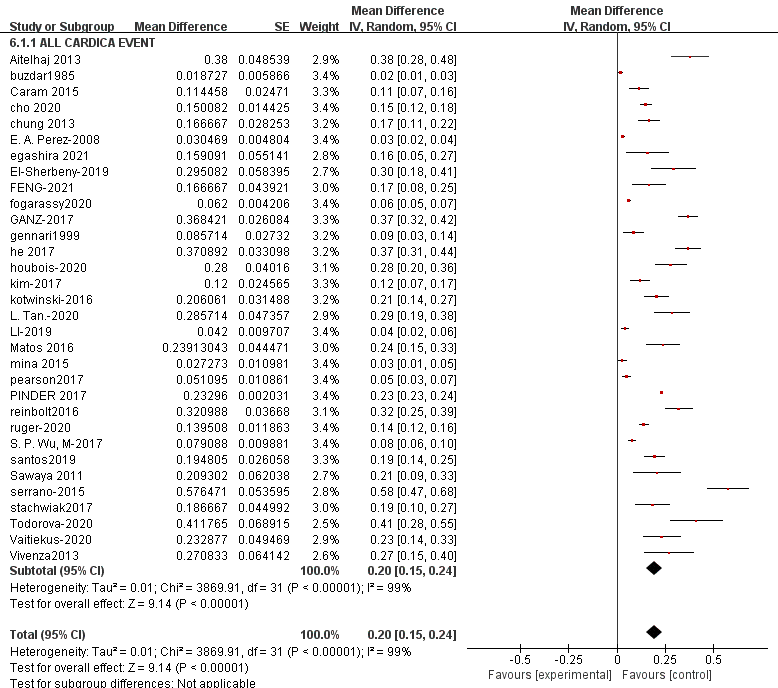


**Figure S1**

Forest plot of the incidence of anthracycline-induced cardiotoxicity. CI, confidence intervals;


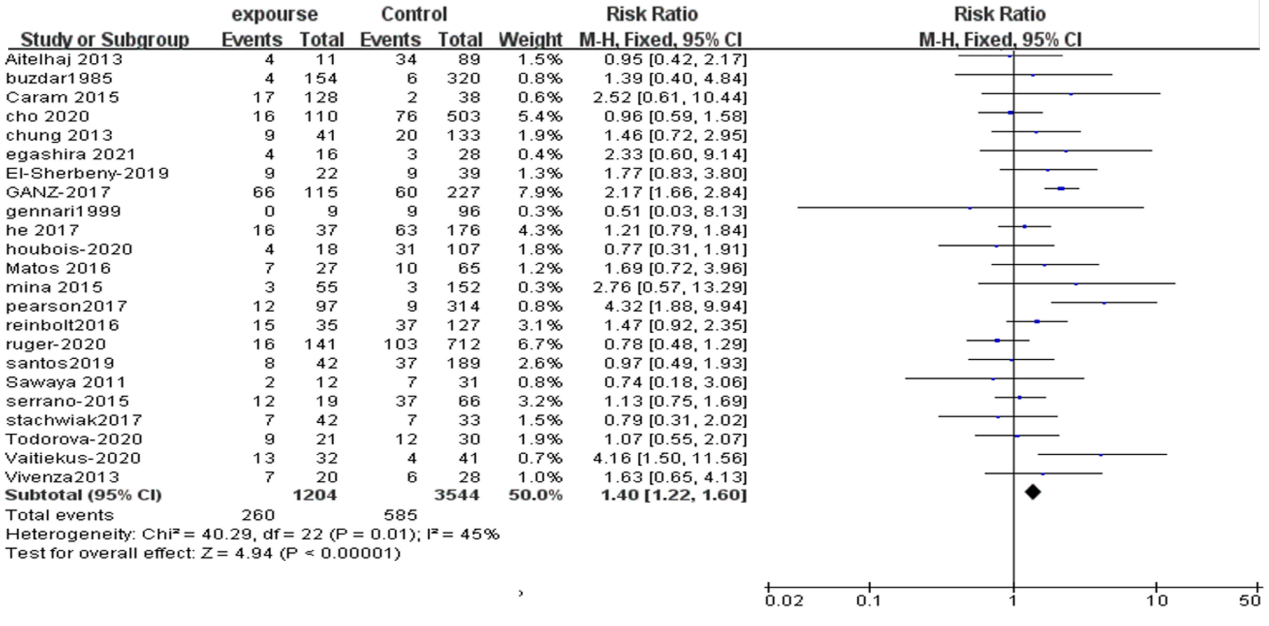
**Figure S2**

Forest plot for the effects of hypertension on the risk of anthracycline-induced cardiotoxicity. CI, confidence intervals; RR,risk ratios.


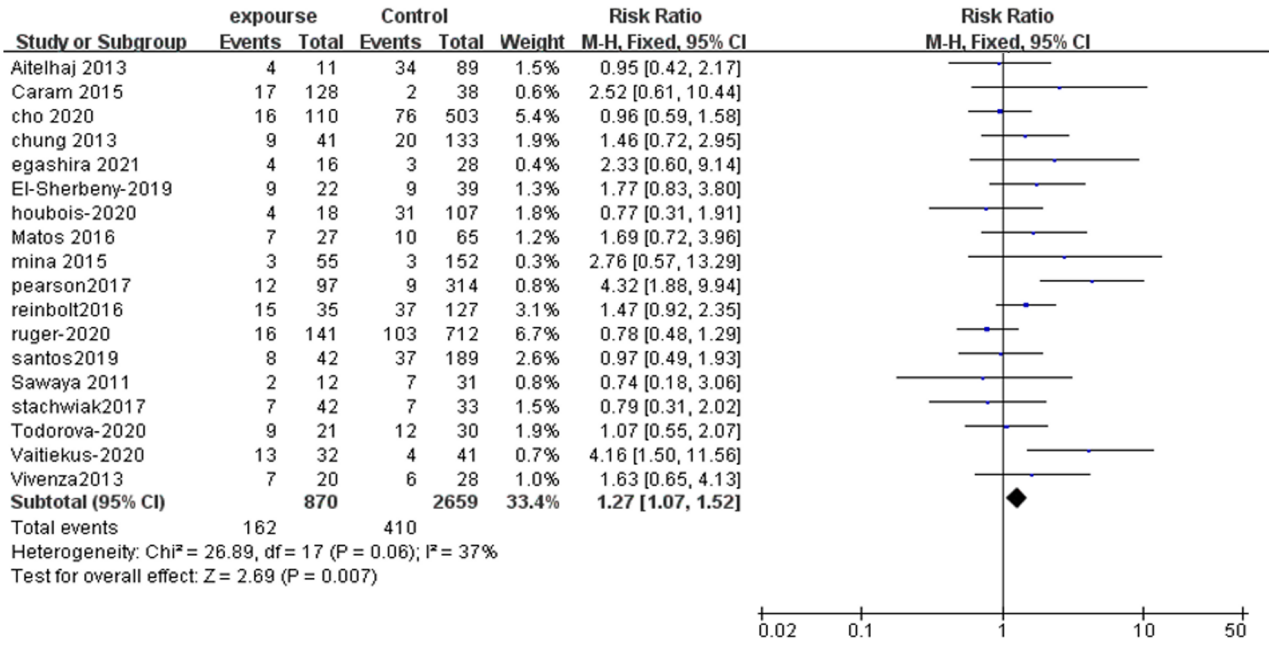


**Figure S3**

Forest plot for the effects of hypertension on the risk of anthracycline-induced cardiotoxicity by LVDF. CI, confidence intervals; RR,risk ratios.


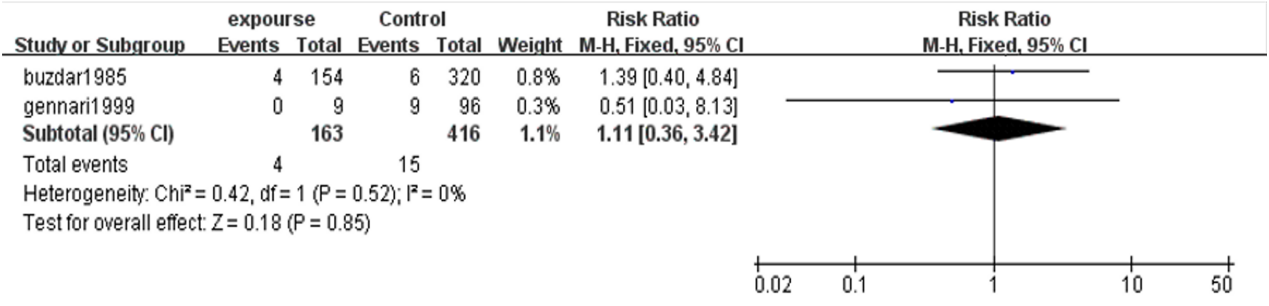


**Figure S4**

Forest plot for the effects of hypertension on the risk of anthracycline-induced cardiotoxicity by CHF. CI, confidence intervals; RR,risk ratios.


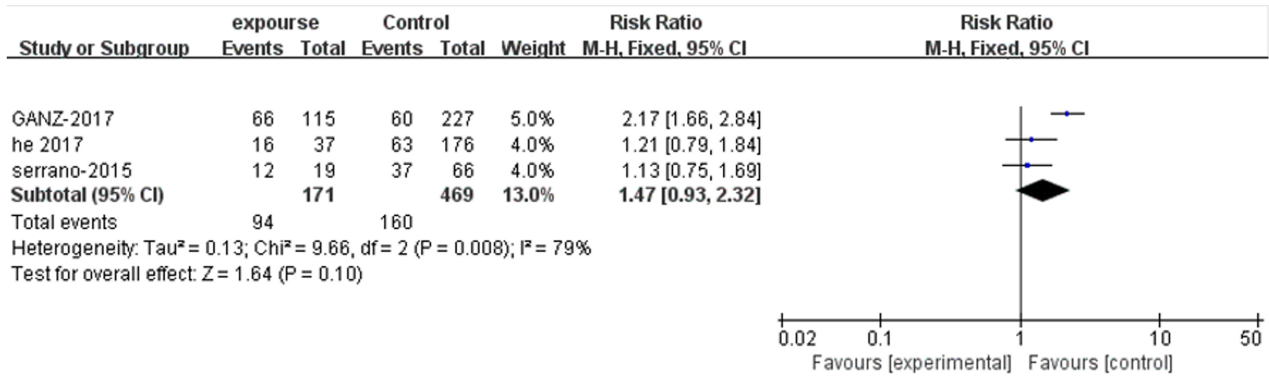


**Figure S5**

Forest plot for the effects of hypertension on the risk of anthracycline-induced cardiotoxicity by Other Cardiac Events. CI, confidence intervals; RR,risk ratios.


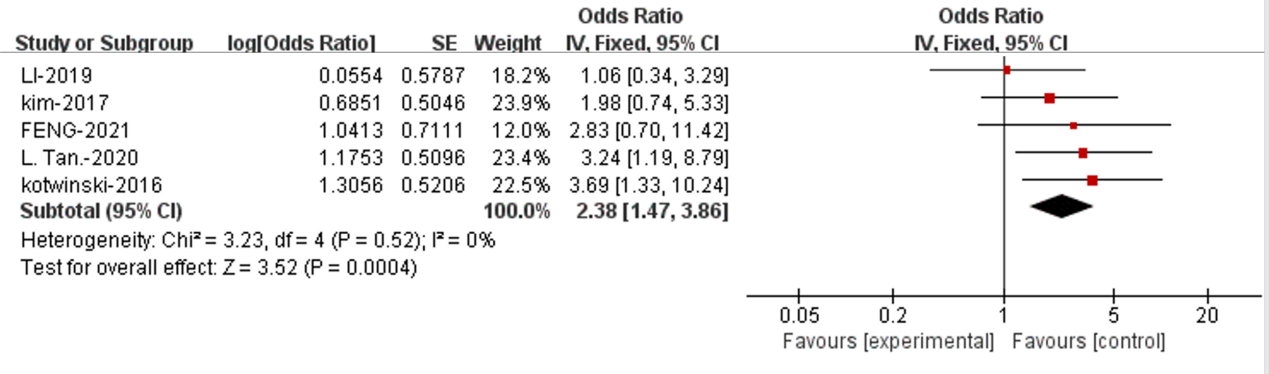


**Figure S6**

Forest plot for the effects of hypertension on the risk of anthracycline-induced cardiotoxicity by LVDF (data was presented with ORs and their 95% CI in relavant stuidies). CI, confidence intervals; OR,odds ratios.


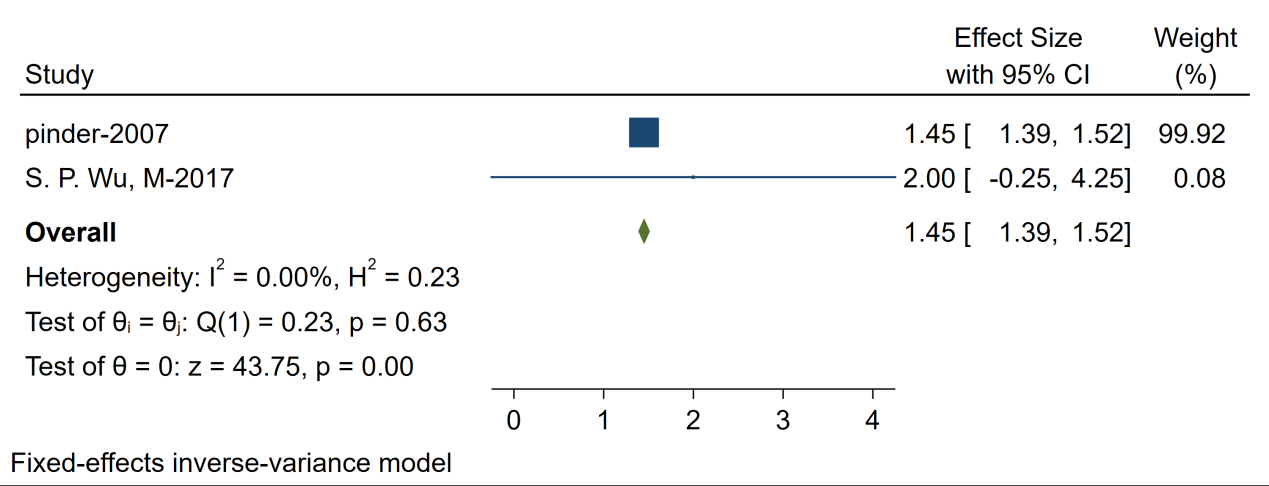


**Figure S7**

Forest plot for the effects of hypertension on the risk of anthracycline-induced cardiotoxicity. CI, confidence intervals; HR, hazard ratio.


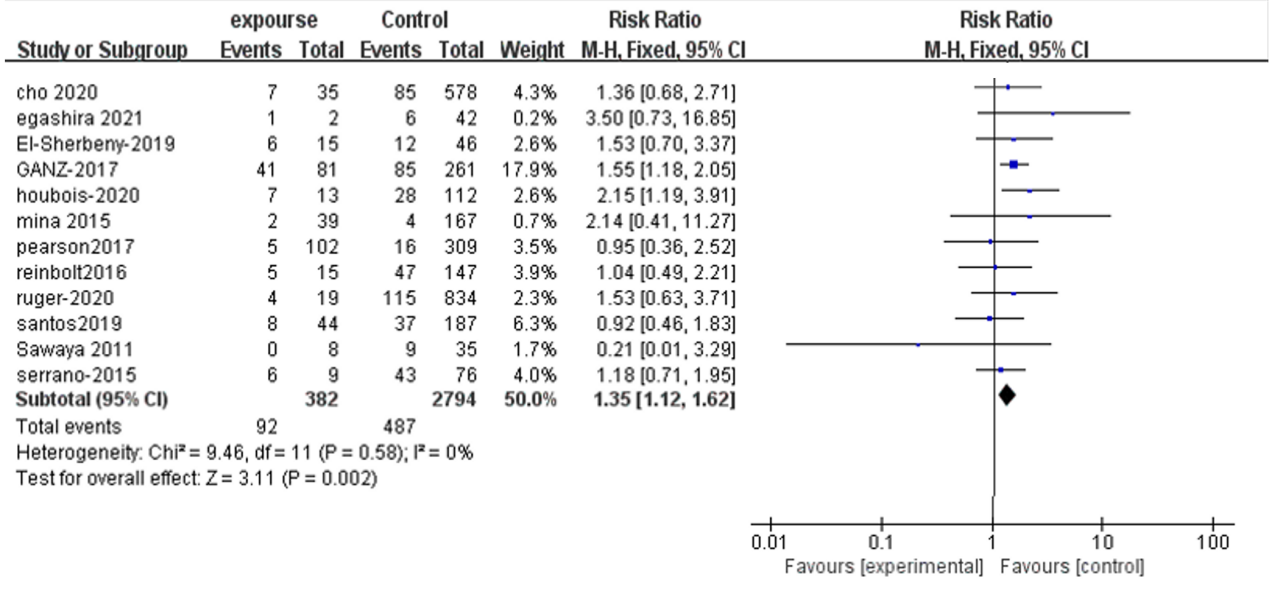


**Figure S8**

Forest plot for the effects of hyperlipidaemia on the risk of anthracycline-induced cardiotoxicity. CI, confidence intervals; RR,risk ratios.


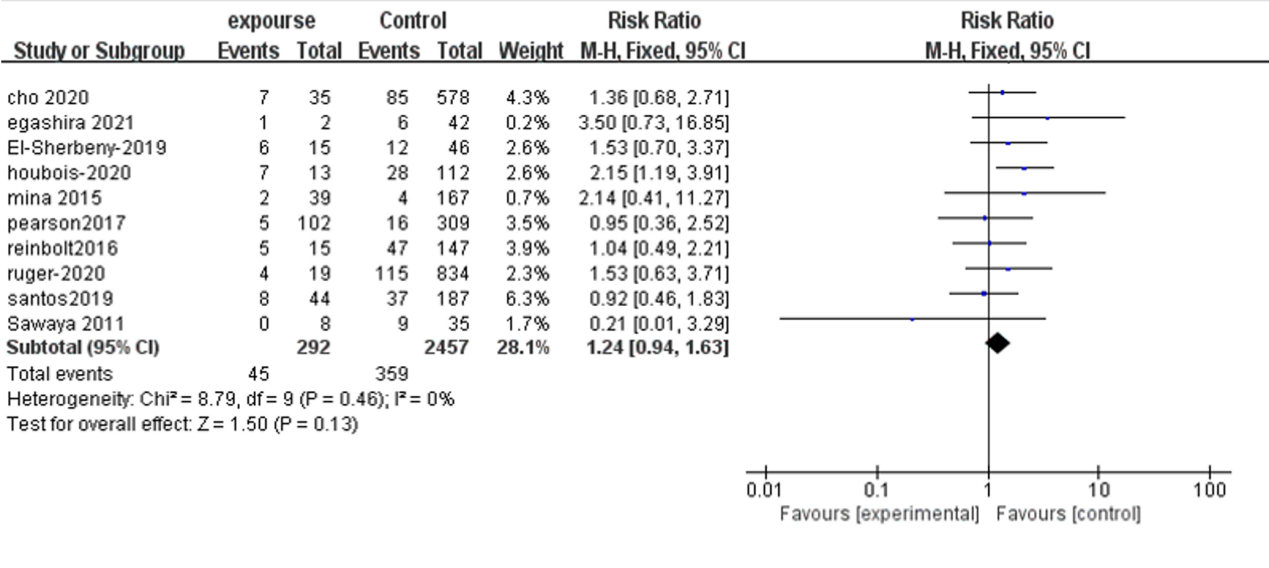


**Figure S9**

Forest plot for the effects of hyperlipidaemia on the risk of anthracycline-induced cardiotoxicity by LVDF. CI, confidence intervals; RR,risk ratios.


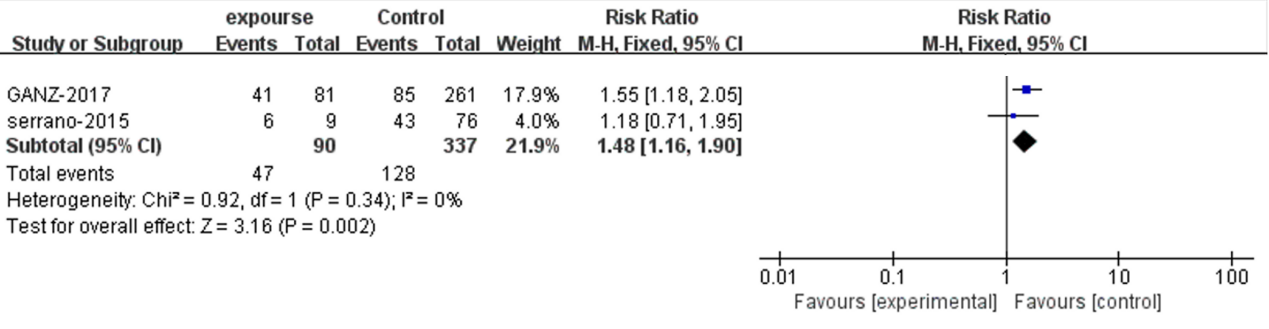


**Figure S10**

Forest plot for the effects of hyperlipidaemia on the risk of anthracycline-induced cardiotoxicity by Other Cardiac Events. CI, confidence intervals; RR,risk ratios.


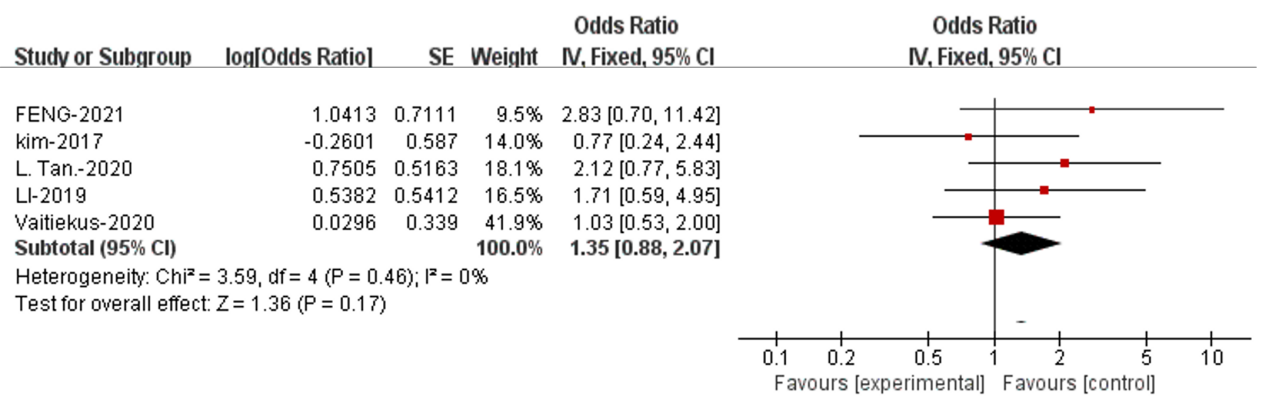


**Figure S11**

Forest plot for the effects of hyperlipidaemia on the risk of anthracycline-induced cardiotoxicity by LVDF (data was presented with ORs and their 95% CI in relavant stuidies).. CI, confidence intervals; OR,odds ratios


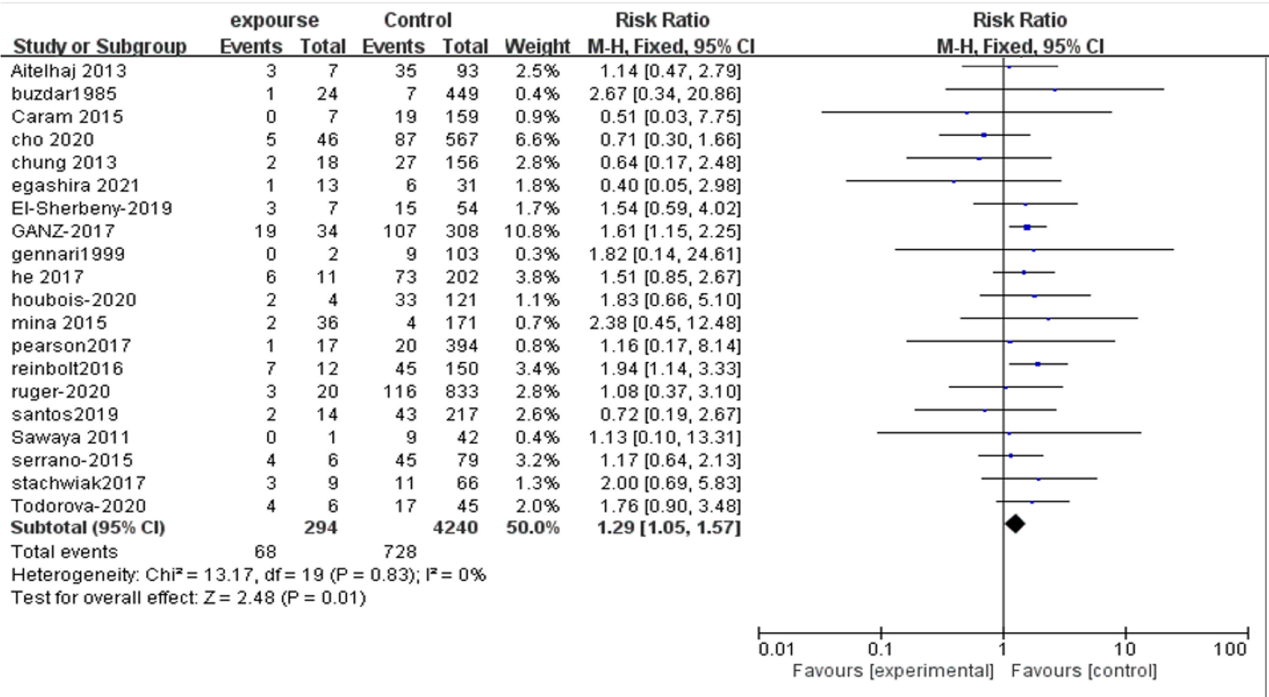


**Figure S12**

Forest plot for the effects of diabetes on the risk of anthracycline-induced cardiotoxicity. CI, confidence intervals; RR,risk ratios


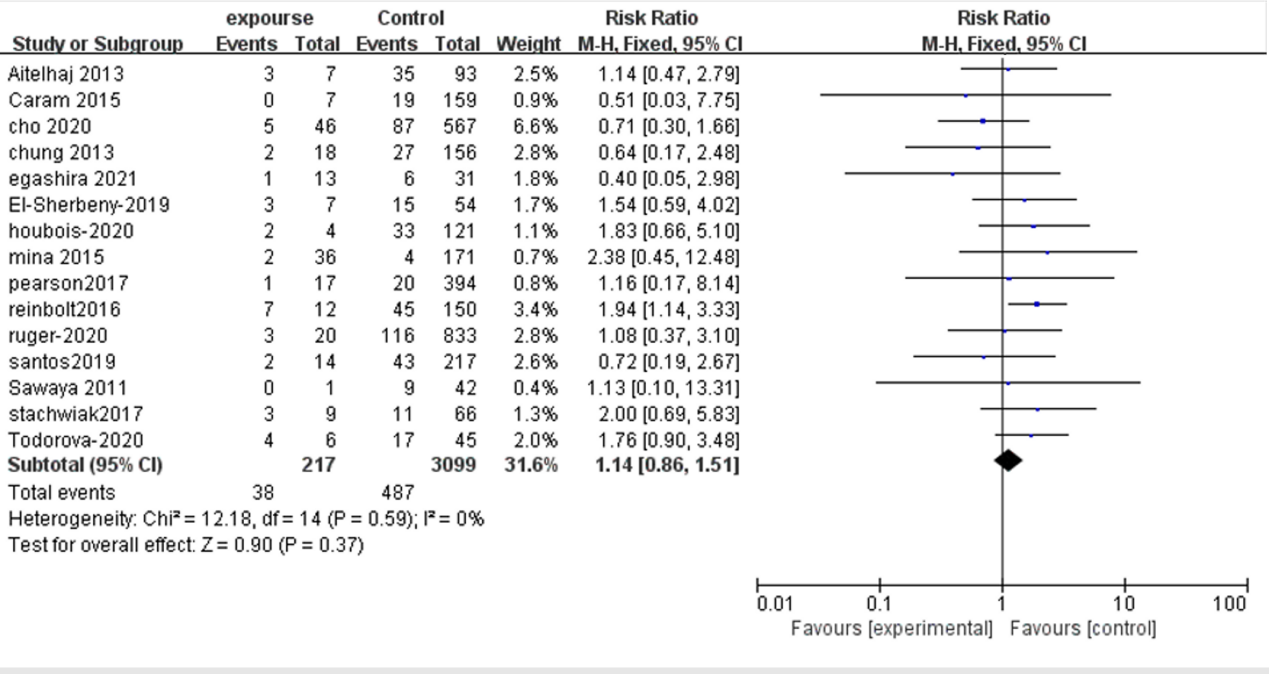


**Figure S13**

Forest plot for the effects of diabetes on the risk of anthracycline-induced cardiotoxicity by LVDF. CI, confidence intervals; RR,risk ratios.


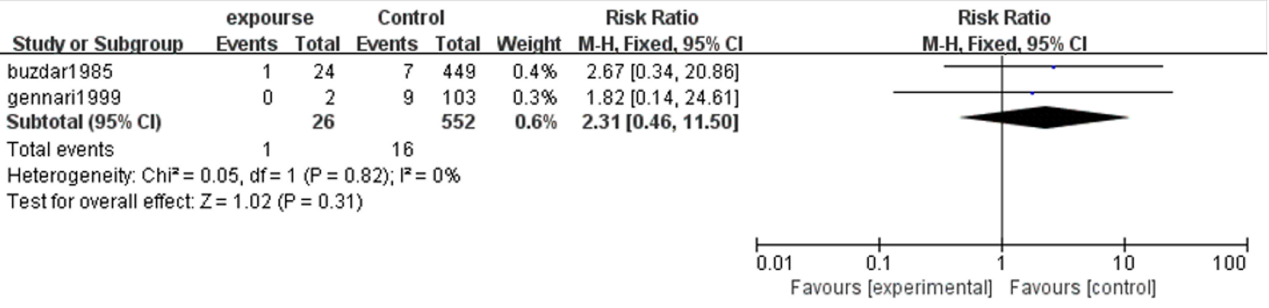


**Figure S14**

Forest plot for the effects of diabetes on the risk of anthracycline-induced cardiotoxicity by CHF. CI, confidence intervals; RR,risk ratios.


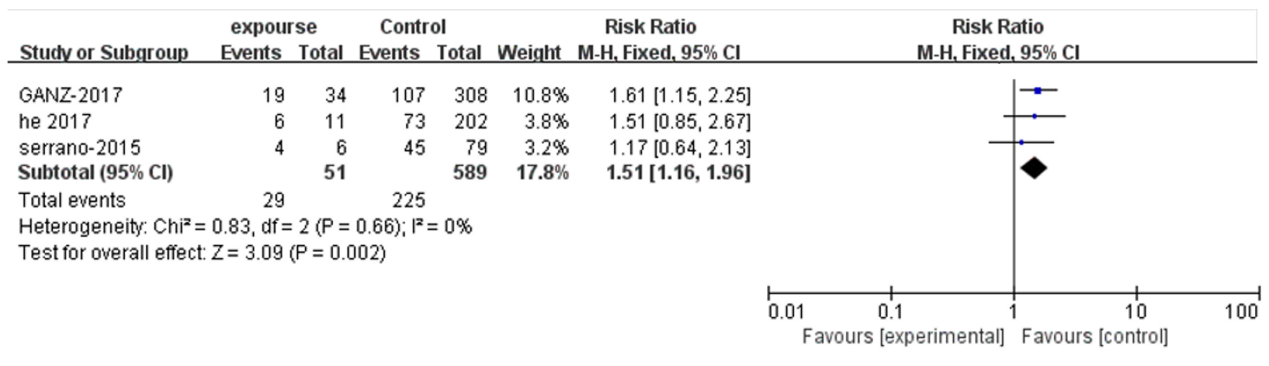


**Figure S15**

Forest plot for the effects of diabetes on the risk of anthracycline-induced cardiotoxicity by Other Cardiac Events. CI, confidence intervals; RR,risk ratios.


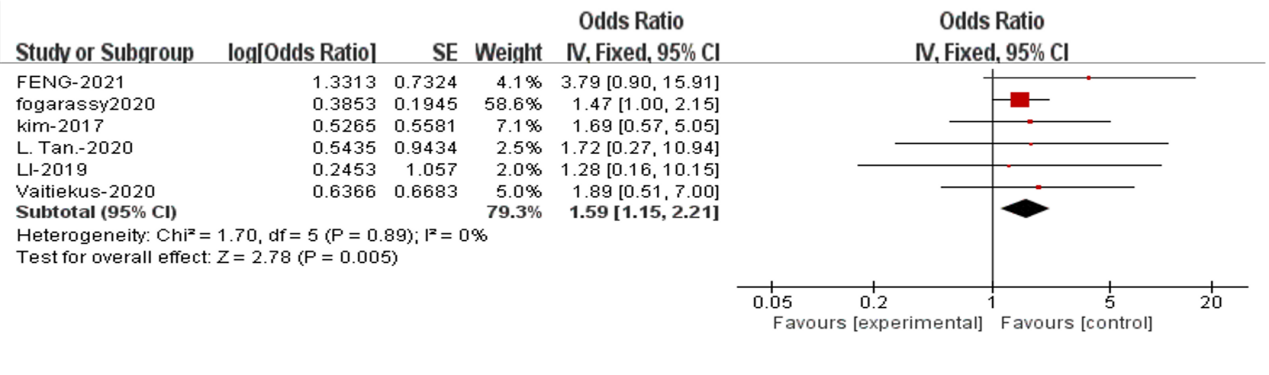


**Figure S16**

Forest plot for the effects of diabetes on the risk of anthracycline-induced cardiotoxicity (data was presented with ORs and their 95% CI in relavant stuidies).. CI, confidence intervals; OR,odds ratios


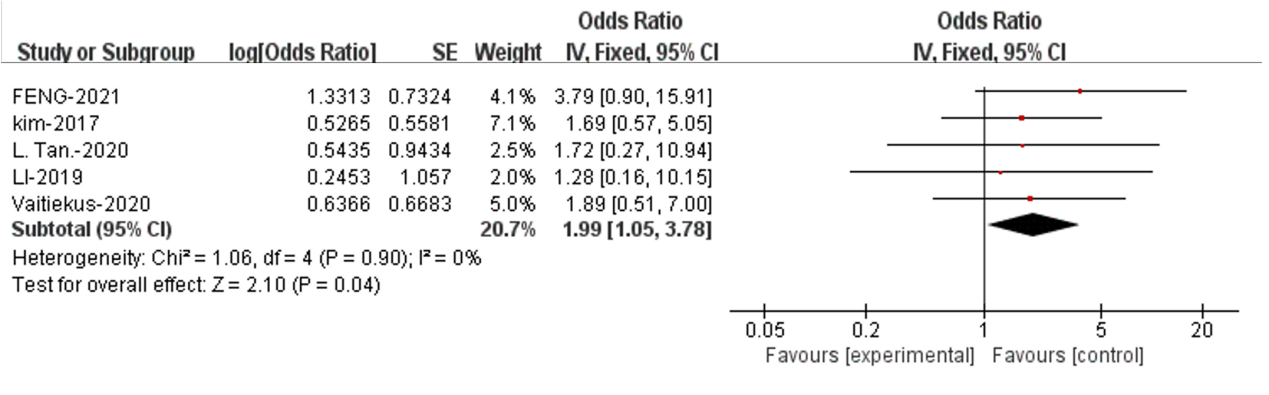


**Figure S17**

Forest plot for the effects of diabetes on the risk of anthracycline-induced cardiotoxicity by LVDF (data was presented with ORs and their 95% CI in relavant stuidies).. CI, confidence intervals; OR,odds ratios


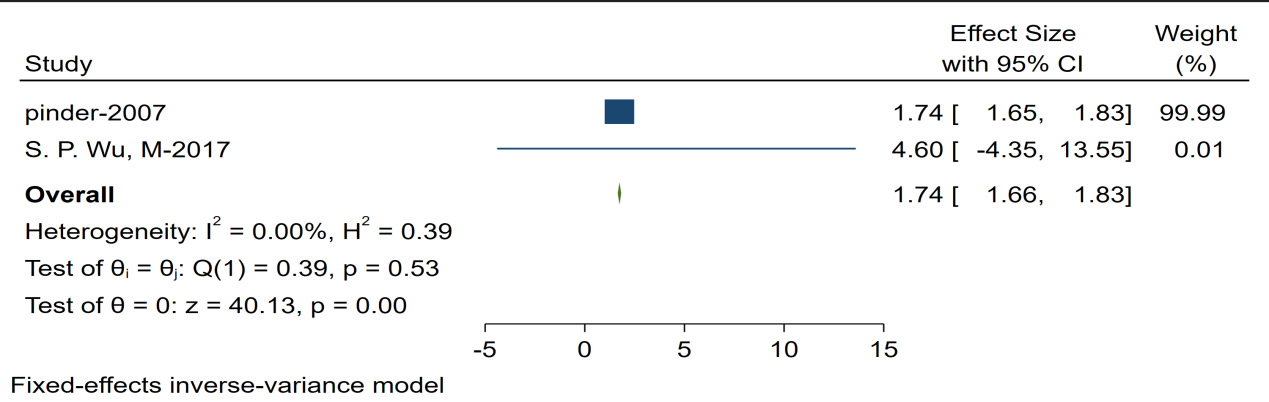
**Figure S18**

Forest plot for the effects of diabetes on the risk of anthracycline-induced cardiotoxicity. CI, confidence intervals; HR, hazard ratio.


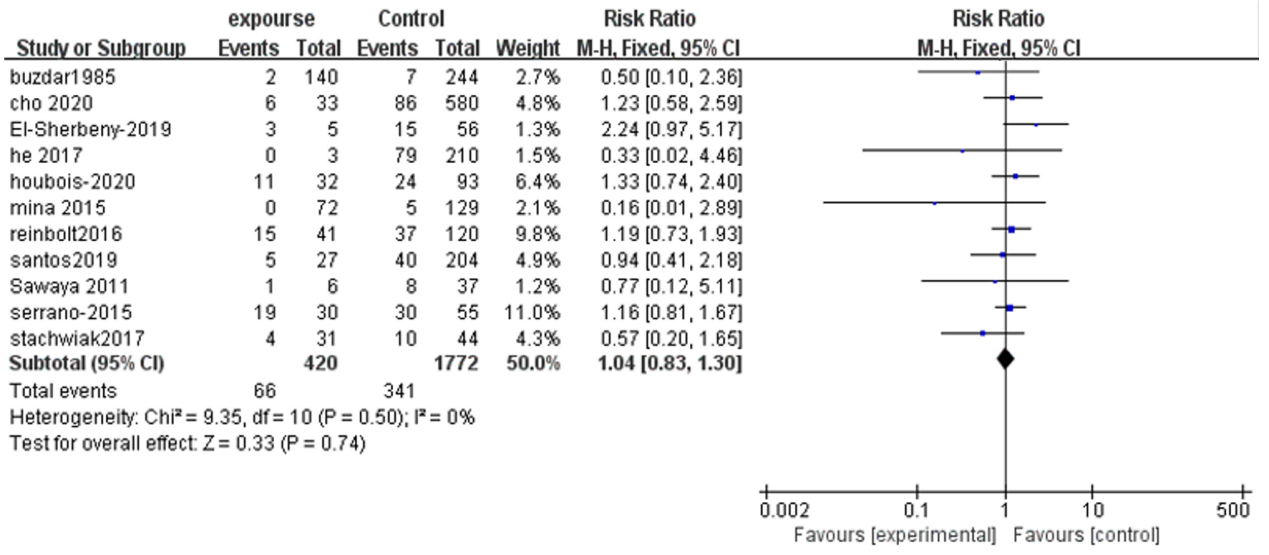


**Figure S19**

Forest plot for the effects of smoke on the risk of anthracycline-induced cardiotoxicity. CI, confidence intervals; RR,risk ratios


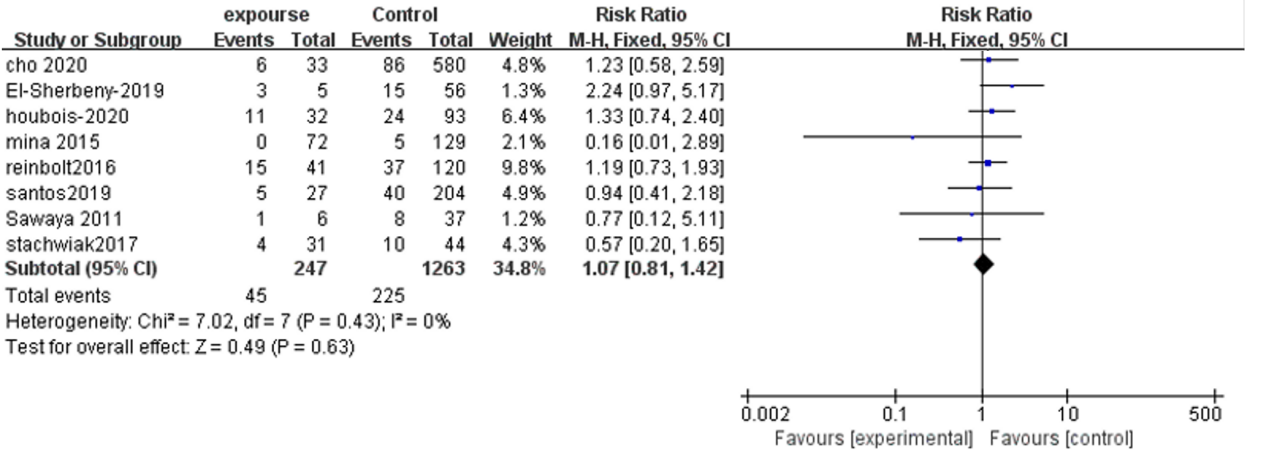


**Figure S20**

Forest plot for the effects of smoke on the risk of anthracycline-induced cardiotoxicity by LVDF. CI, confidence intervals; RR,risk ratios.


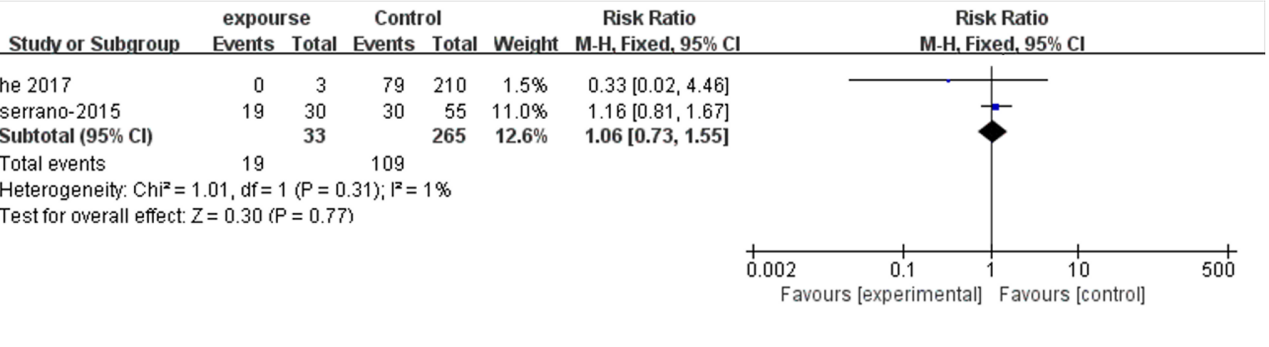


**Figure S21**

Forest plot for the effects of diabetes on the risk of anthracycline-induced cardiotoxicity by Other Cardiac Events. CI, confidence intervals; RR,risk ratios


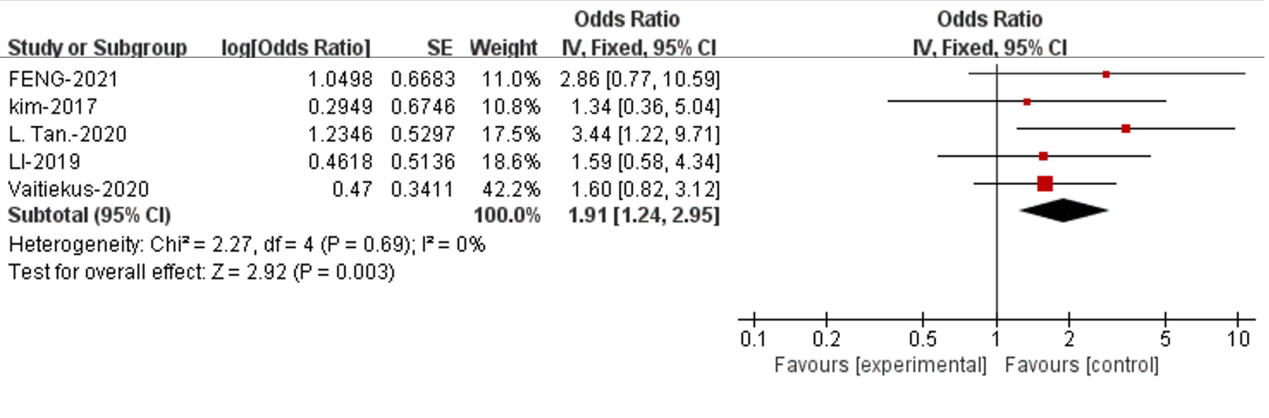


**Figure S22**

Forest plot for the effects of diabetes on the risk of anthracycline-induced cardiotoxicity by LVDF (data was presented with ORs and their 95% CI in relavant stuidies).. CI, confidence intervals; OR,odds ratios


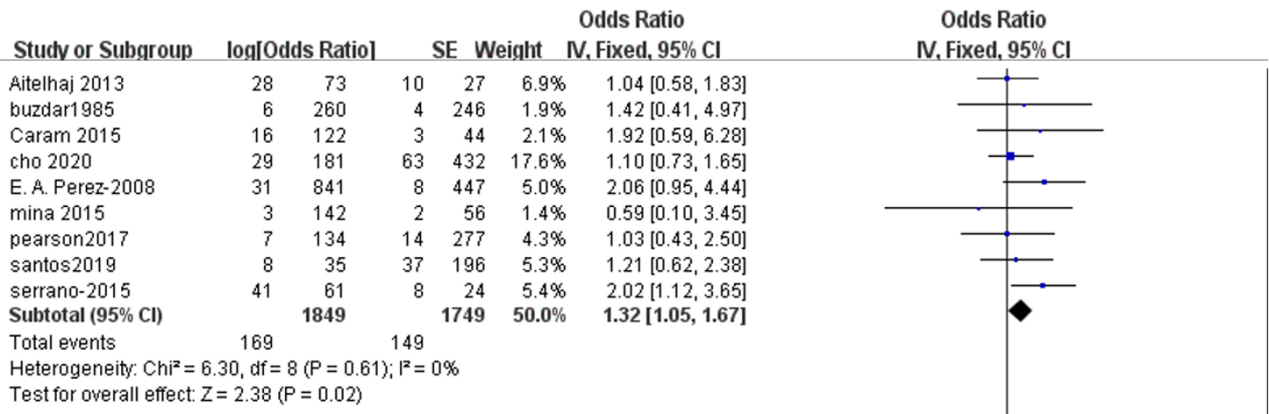
**Figure S23**

Forest plot for the effects of obesity on the risk of anthracycline-induced cardiotoxicity. CI, confidence intervals; RR,risk ratios


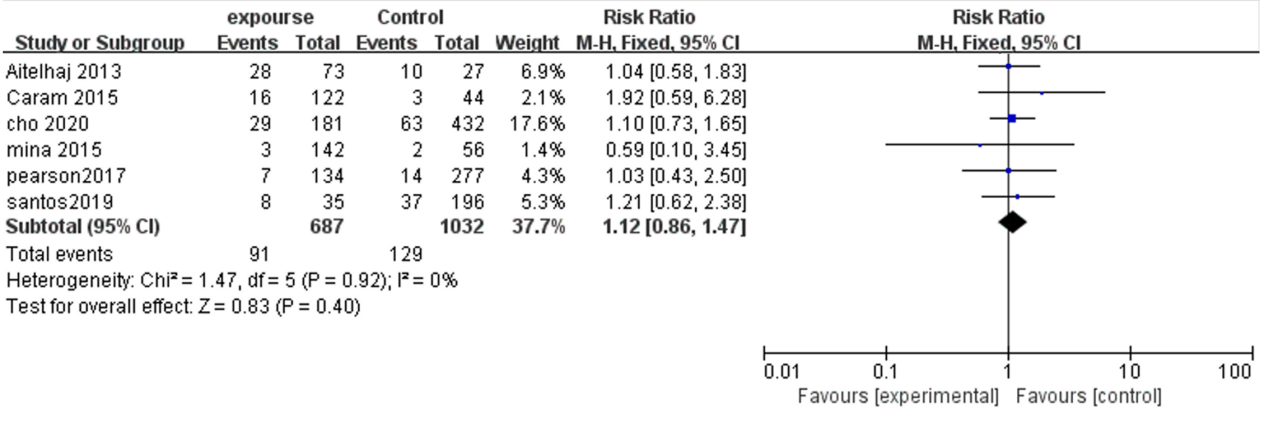
**Figure S24**

Forest plot for the effects of obesity on the risk of anthracycline-induced cardiotoxicity by LVDF. CI, confidence intervals; RR,risk ratios.


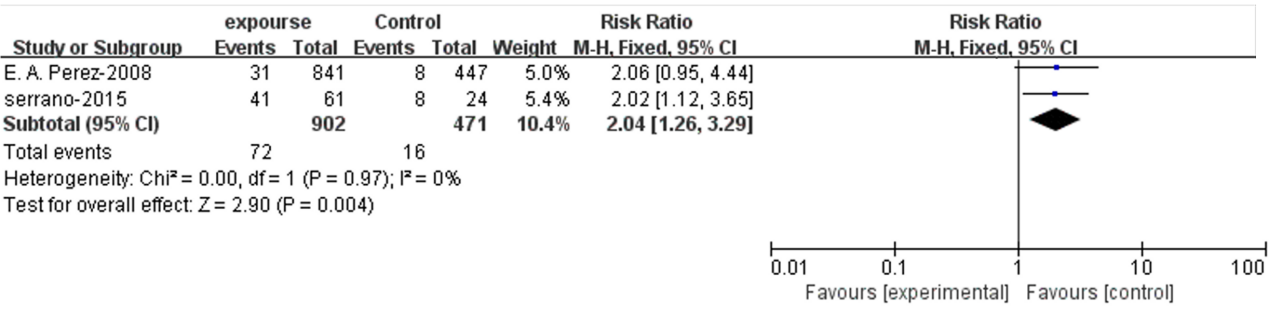


**Figure S25**

Forest plot for the effects of obesity on the risk of anthracycline-induced cardiotoxicity by other cardiac event. CI, confidence intervals; RR,risk ratios.


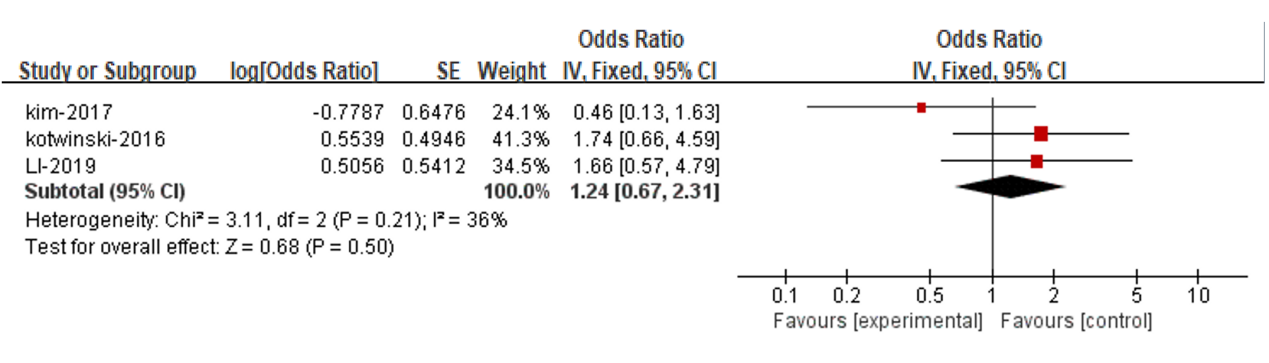
**Figure S26**

Forest plot for the effects of obesity on the risk of anthracycline-induced cardiotoxicity by LVDF (data was presented with ORs and their 95% CI in relavant stuidies).. CI, confidence intervals; OR,odds ratios


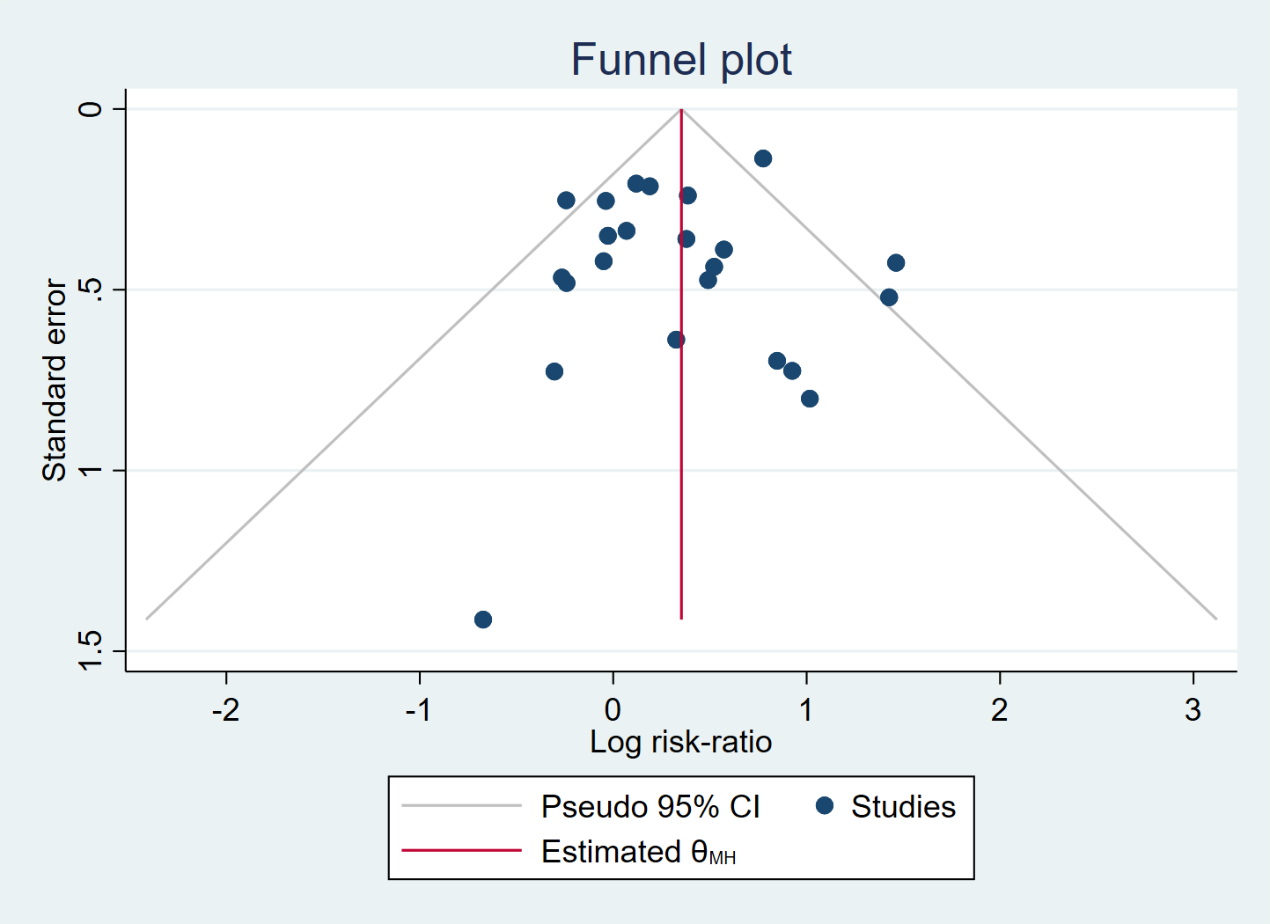


**Figure S27**

Funnel plot with pseudo 95% CIs. Publication bias in studies on the associations of hypertension (23 studies) with cardiotoxicity of anthracyclines. OR, odds ratio


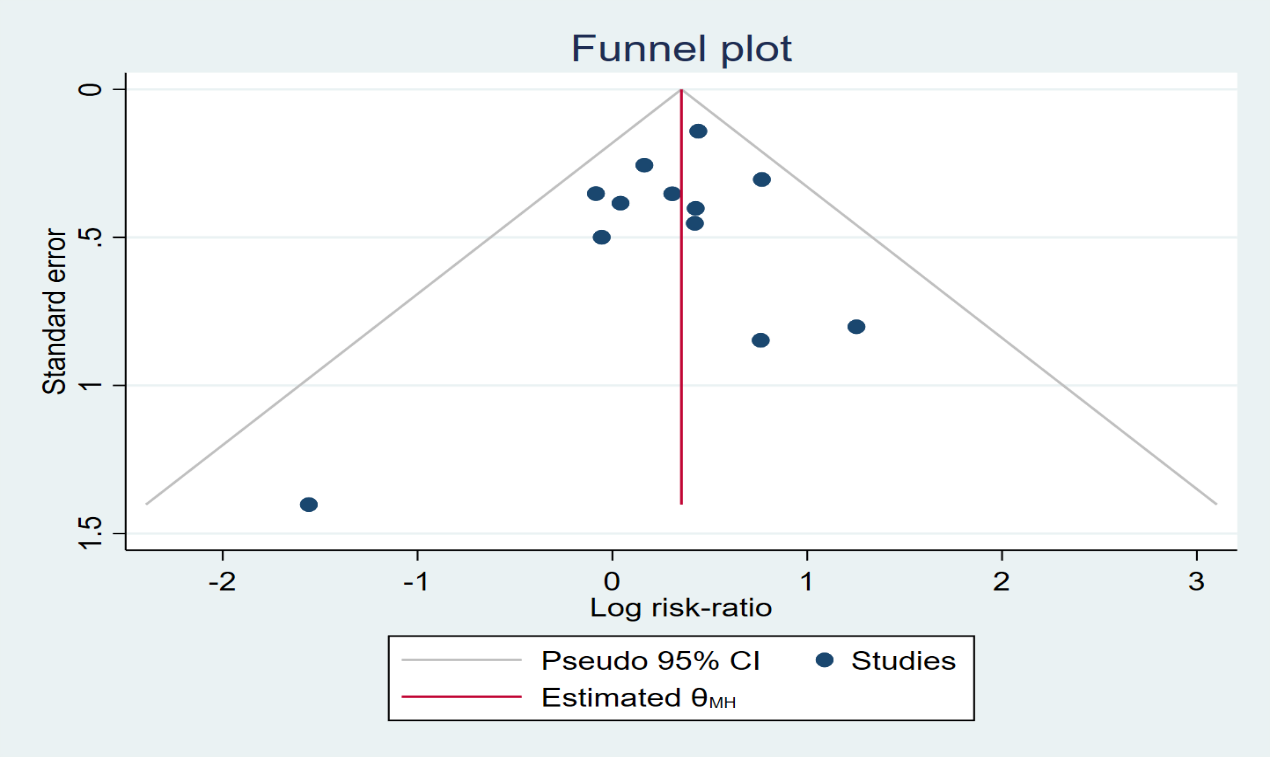


**Figure S28**

Funnel plot with pseudo 95% CIs. Publication bias in studies on the associations of hyperlipidaemia (12 studies) with cardiotoxicity of anthracyclines. OR, odds ratio


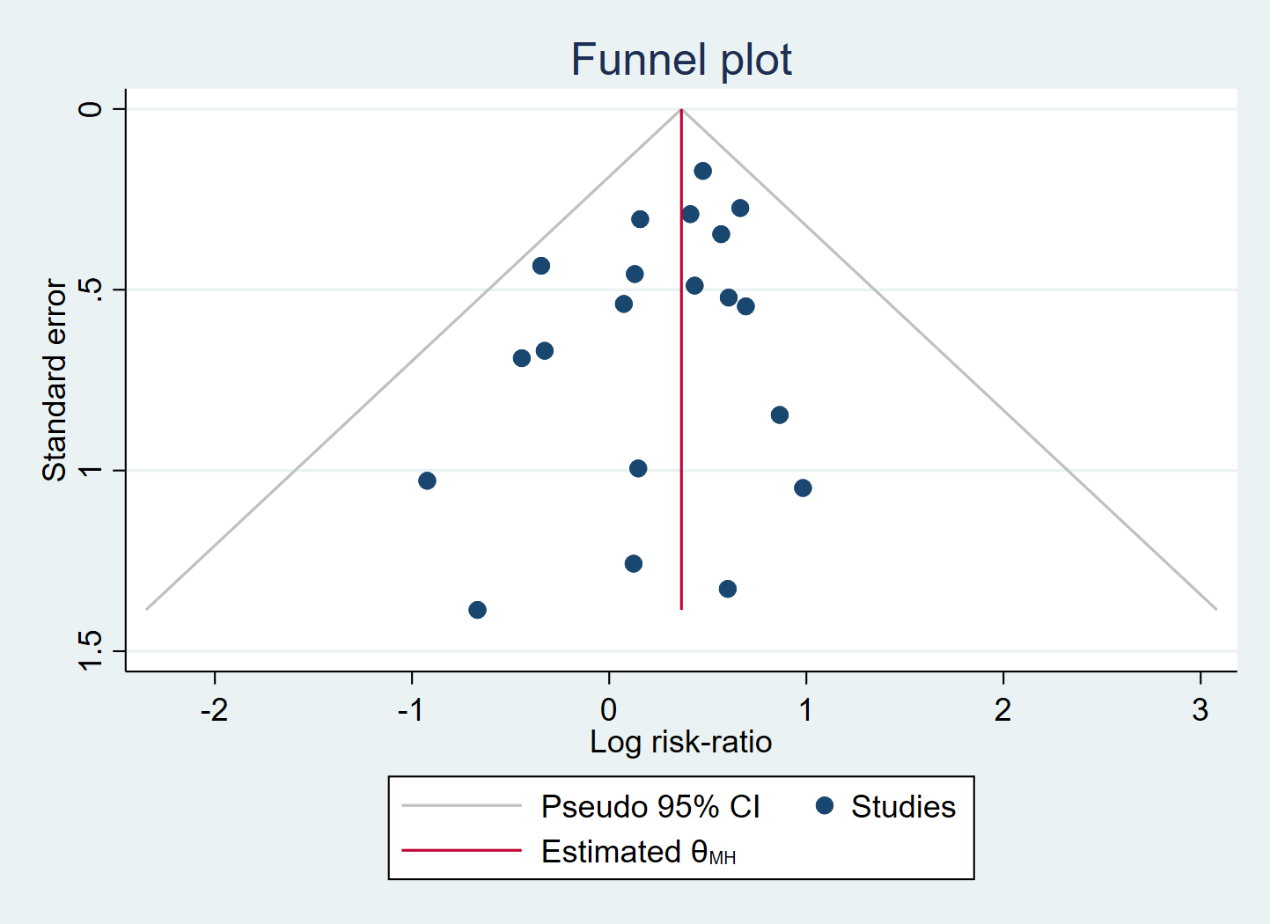


**Figure S29**

Funnel plot with pseudo 95% CIs. Publication bias in studies on the associations of diabetes (20 studies) with cardiotoxicity of anthracyclines. OR, odds ratio


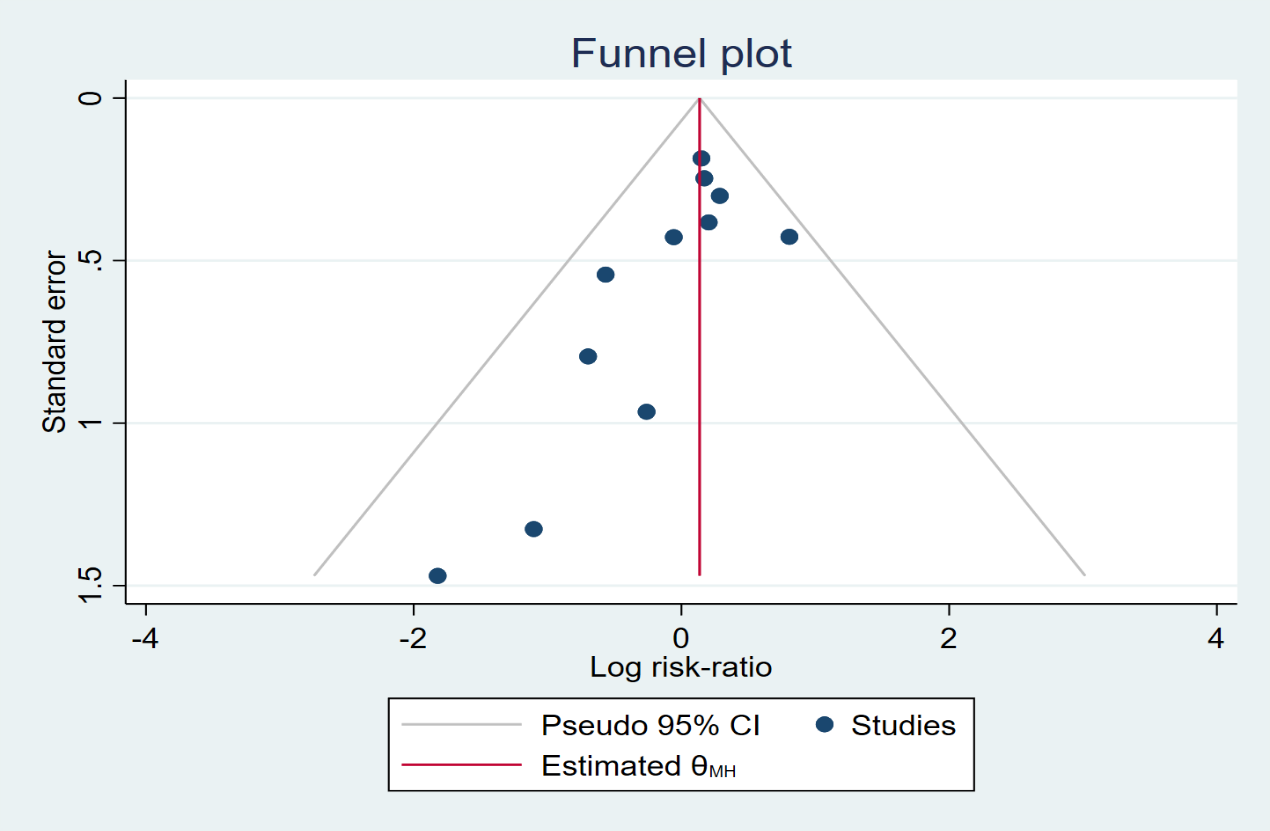


**Figure S30**

Funnel plot with pseudo 95% CIs. Publication bias in studies on the associations of smoke (11 studies) with cardiotoxicity of anthracyclines. OR, odds ratio


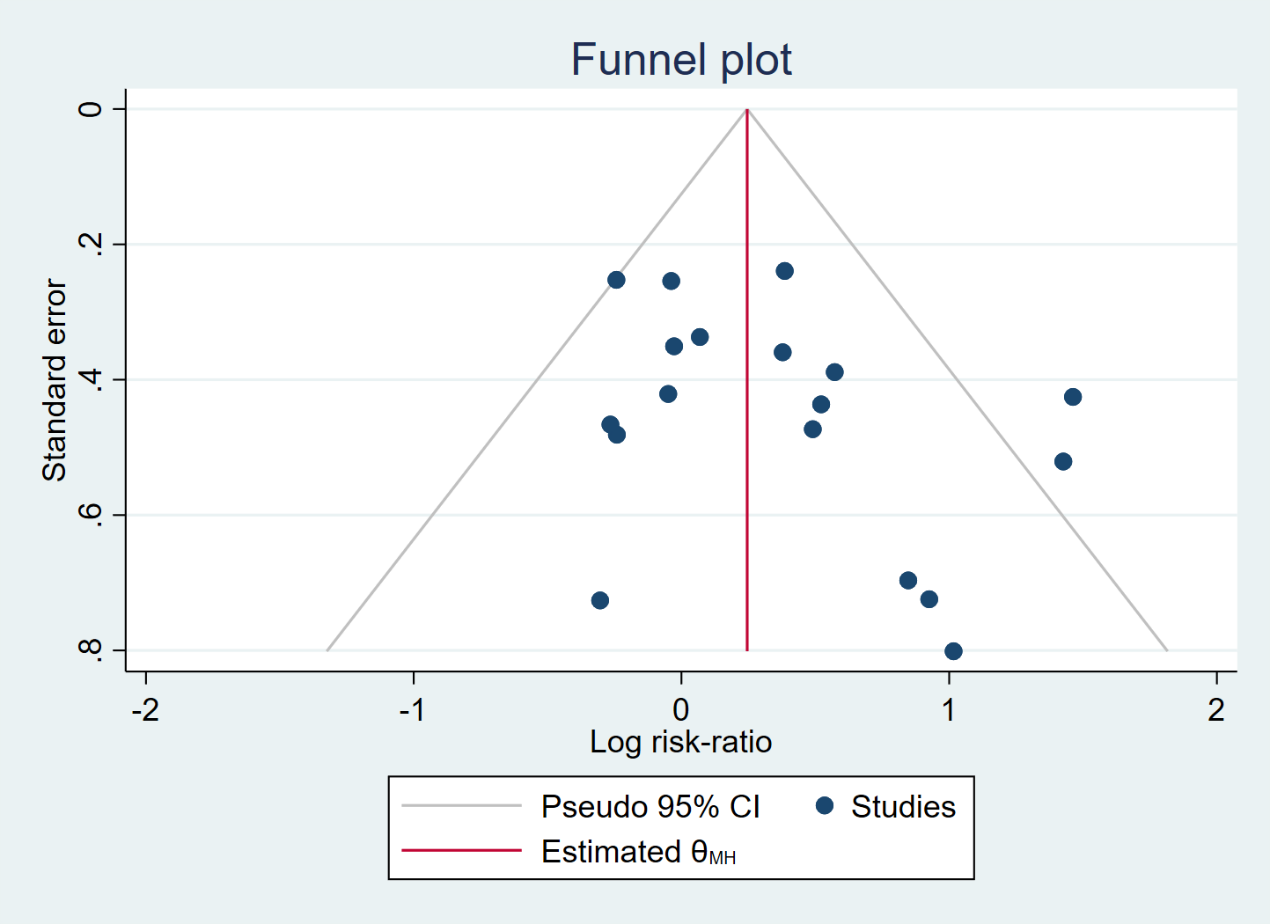


**Figure S31**

Funnel plot with pseudo 95% CIs. Publication bias in studies on the associations of hypertension (18 studies) with cardiotoxicity of anthracyclines by LVDF. OR, odds ratio


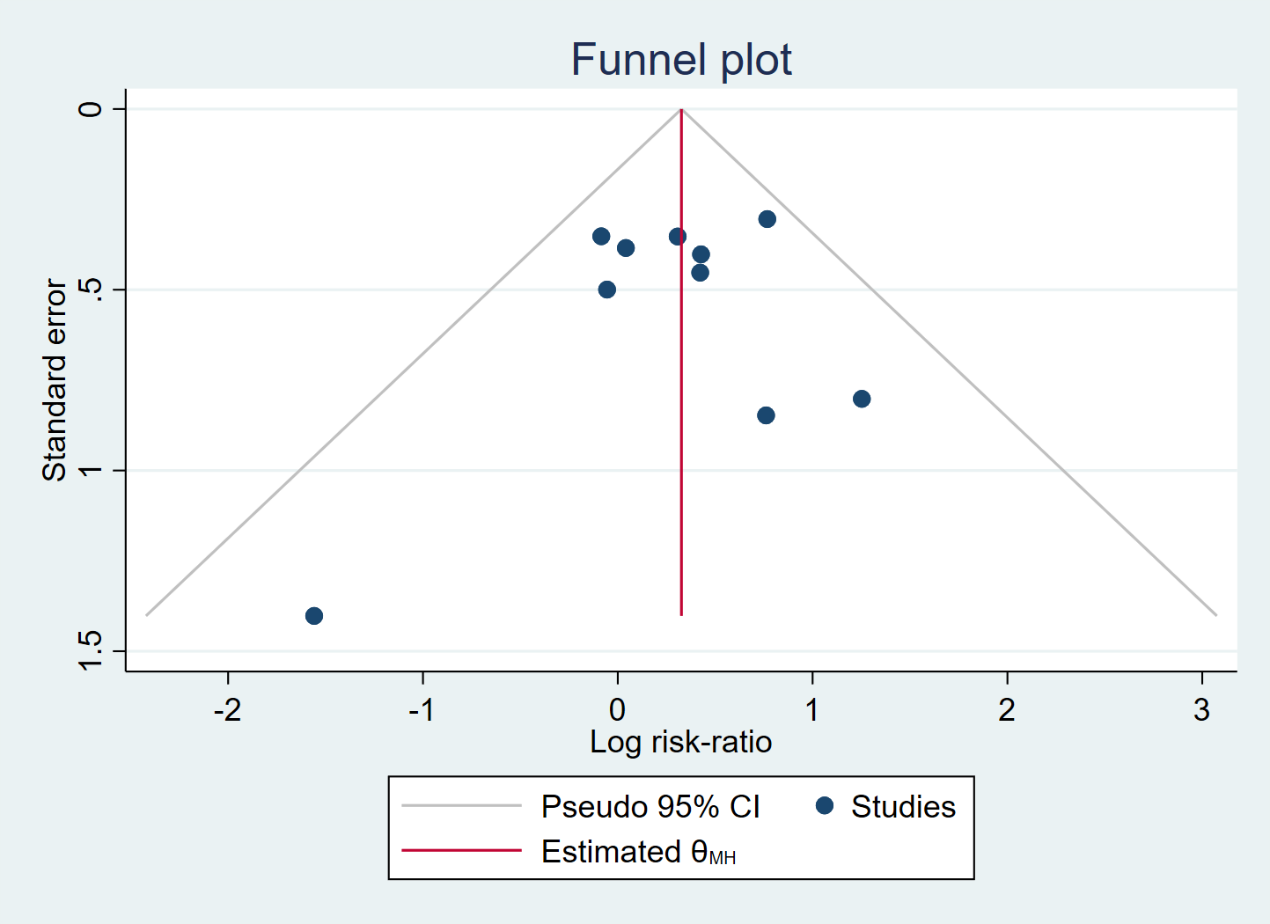


**Figure S32**

Funnel plot with pseudo 95% CIs. Publication bias in studies on the associations of hyperlipidaemia (10 studies) with cardiotoxicity of anthracyclines by LVDF. OR, odds ratio


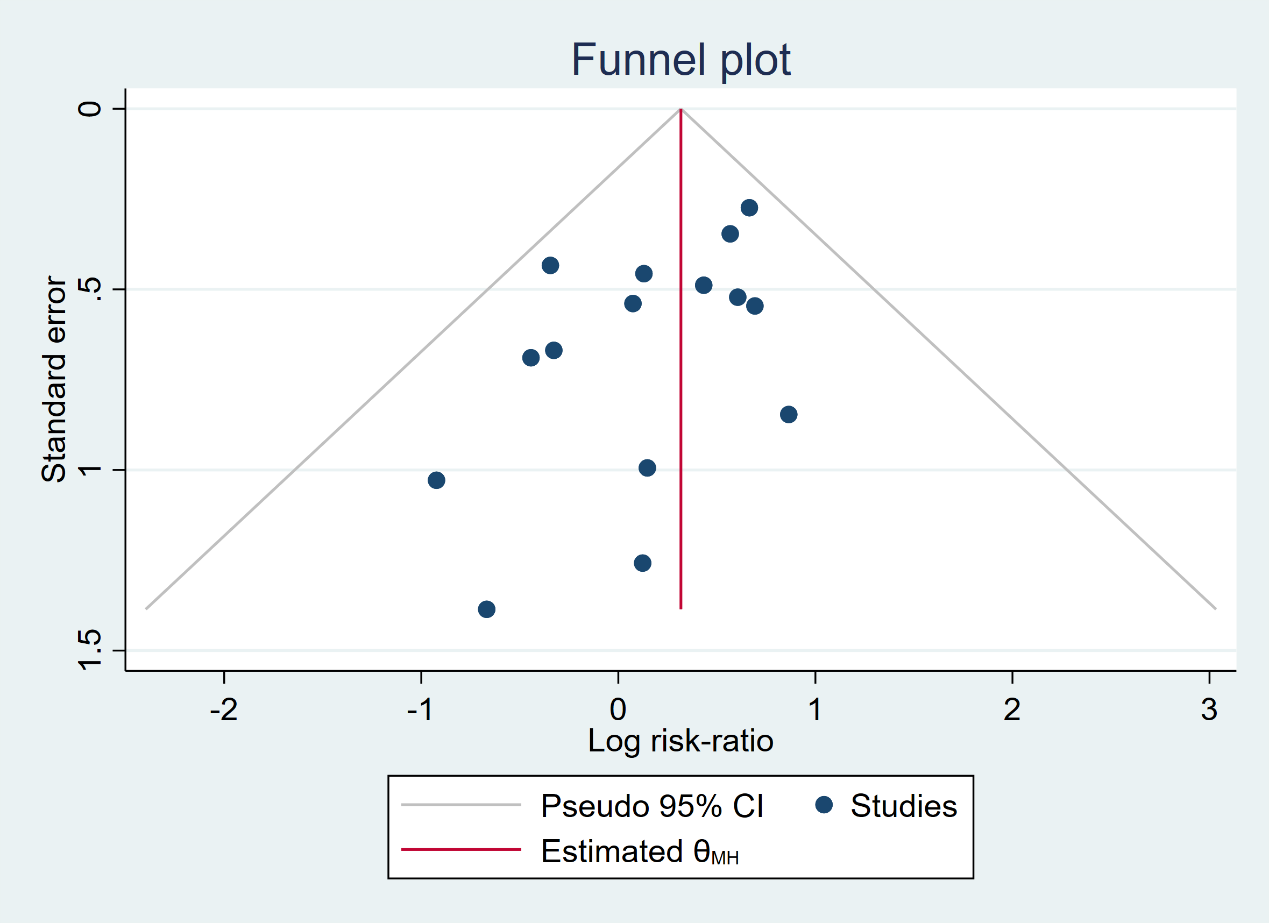


**Figure S33**

Funnel plot with pseudo 95% CIs. Publication bias in studies on the associations of diabetes (15 studies) with cardiotoxicity of anthracyclines by LVDF. OR, odds ratio


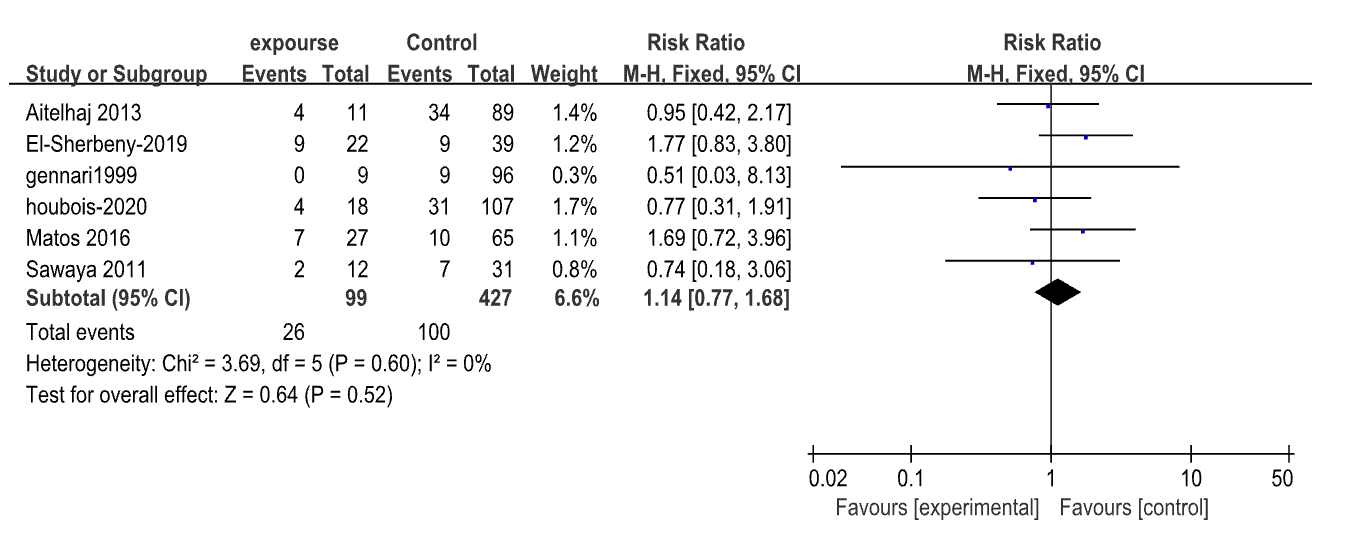


**Figure S34**

Forest plot for the effects of hypertension on the risk of anthracycline-induced cardiotoxicity in Her2+ breast cancer patients. CI, confidence intervals; RR,risk ratios.


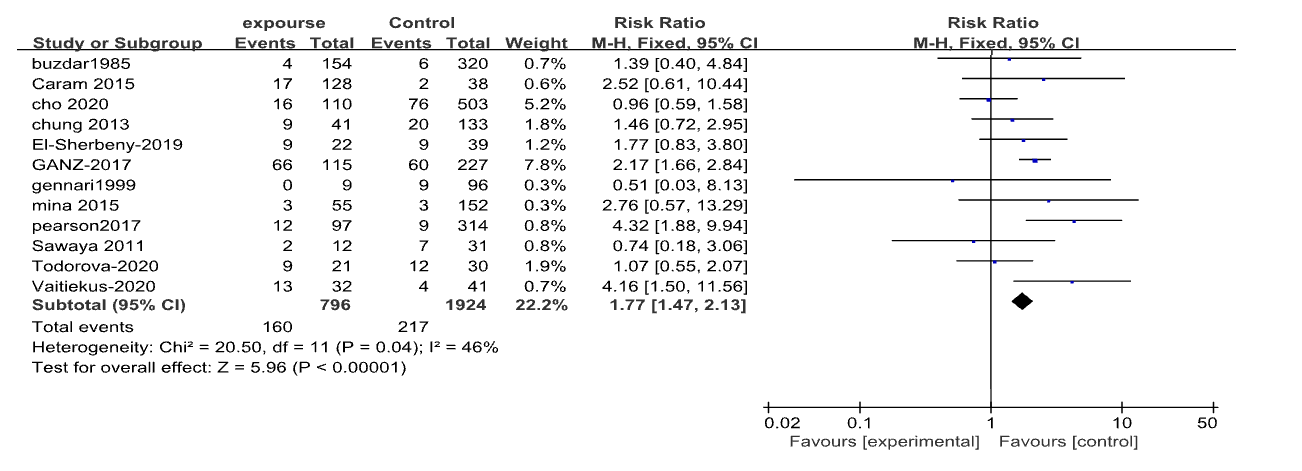


**Figure S35**

Forest plot for the effects of hypertension on the risk of doxorubicin-induced cardiotoxicity. CI, confidence intervals; RR,risk ratios.


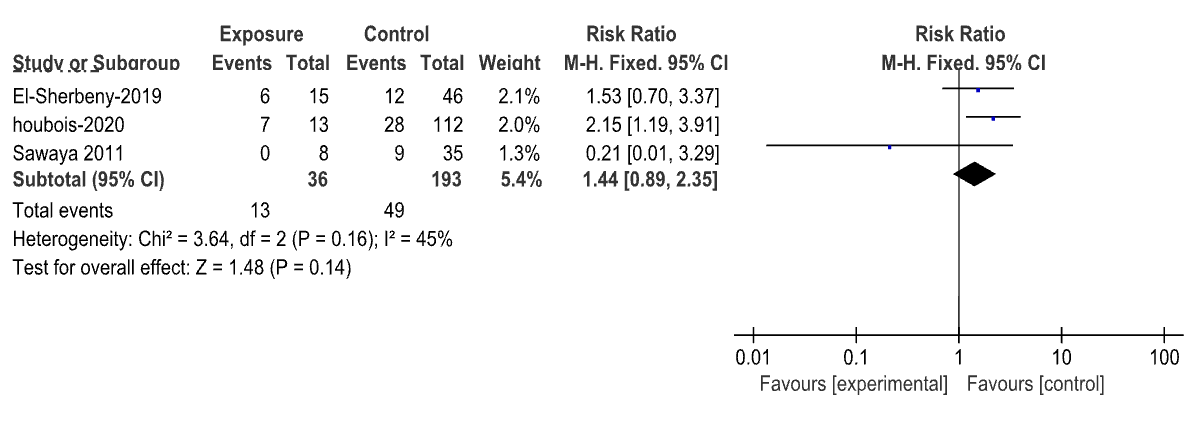


**Figure S36**

Forest plot for the effects of hyperlipidaemia on the risk of anthracycline-induced cardiotoxicity in Her2+ breast cancer patients. CI, confidence intervals; RR,risk ratios.


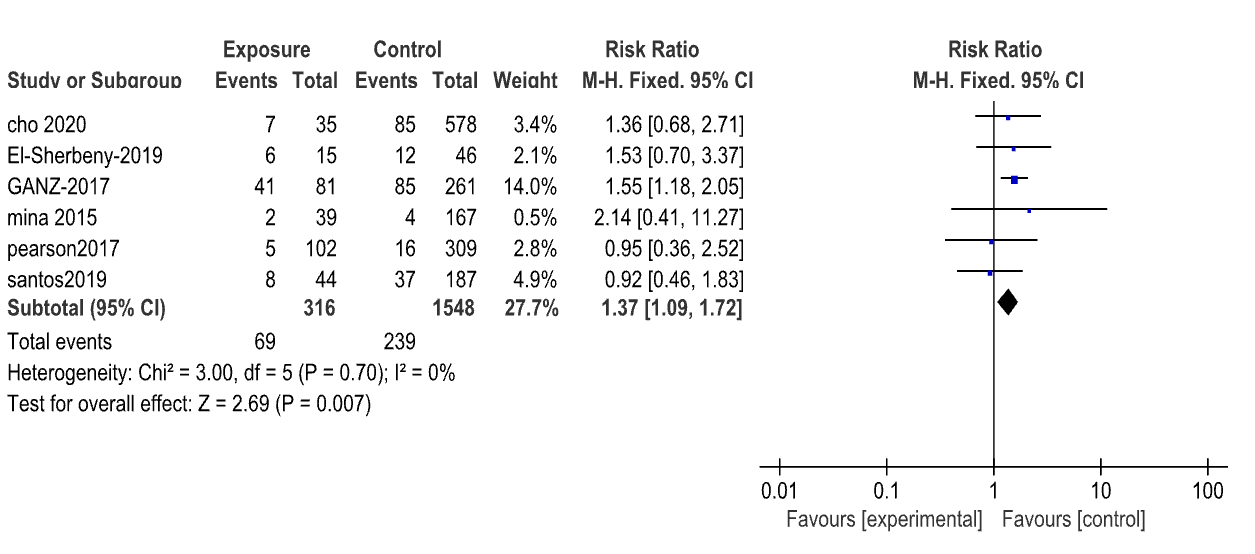


**Figure S37**

Forest plot for the effects of hyperlipidaemia on the risk of doxorubicin-induced cardiotoxicity. CI, confidence intervals; RR,risk ratios.


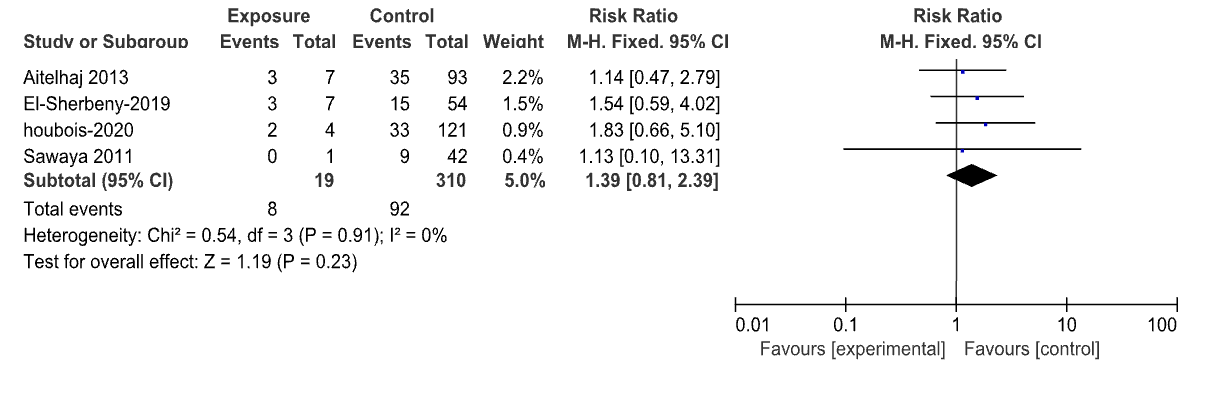


**Figure S38**

Forest plot for the effects of diabetes on the risk of anthracycline-induced cardiotoxicity in Her2+ breast cancer patients. CI, confidence intervals; RR,risk ratios.


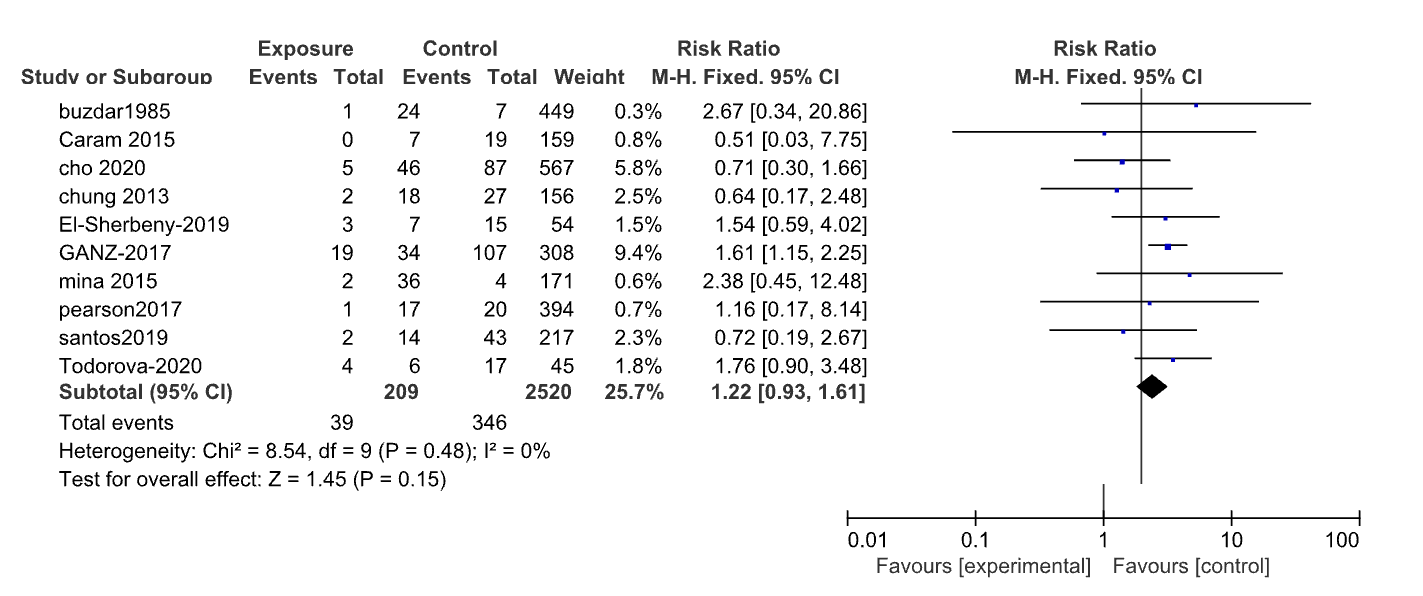


**Figure S39**

Forest plot for the effects of diabetes on the risk of doxorubicin-induced cardiotoxicity. CI, confidence intervals; RR,risk ratios.


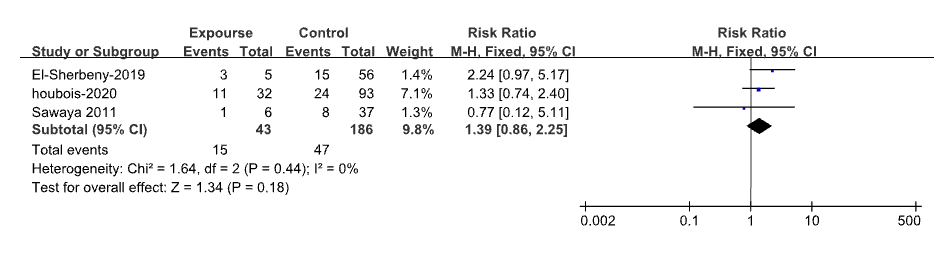


**Figure S40**

Forest plot for the effects of smoke on the risk of anthracycline-induced cardiotoxicity in Her2+ breast cancer patients. CI, confidence intervals; RR,risk ratios.


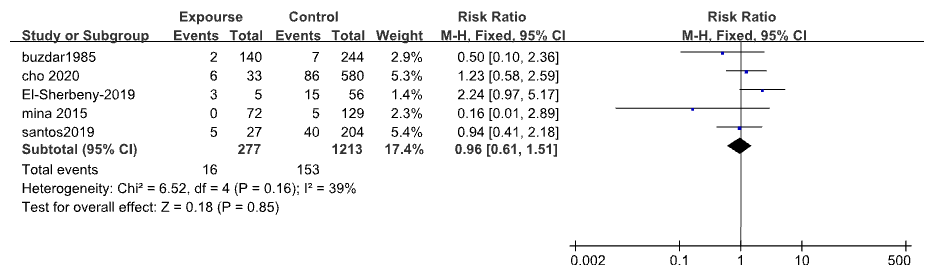


**Figure S41**

Forest plot for the effects of smoke on the risk of doxorubicin-induced cardiotoxicity. CI, confidence intervals; RR,risk ratios.


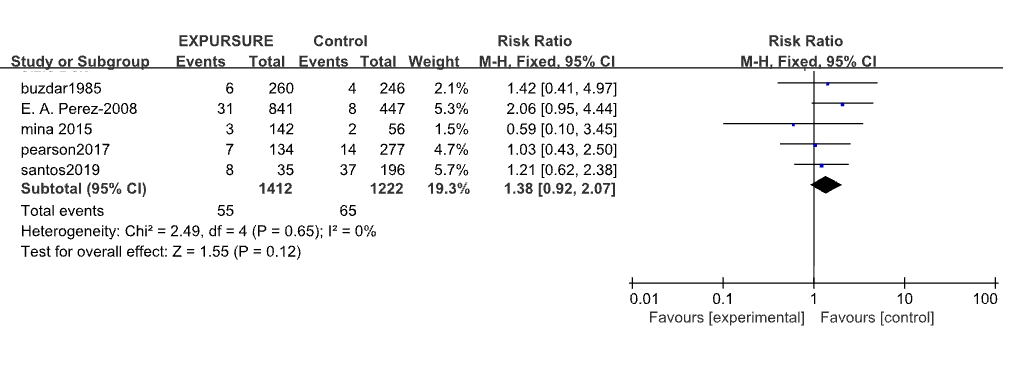


**Figure S42**

Forest plot for the effects of obesity on the risk of doxorubicin-induced cardiotoxicity. CI, confidence intervals; RR,risk ratios.

**
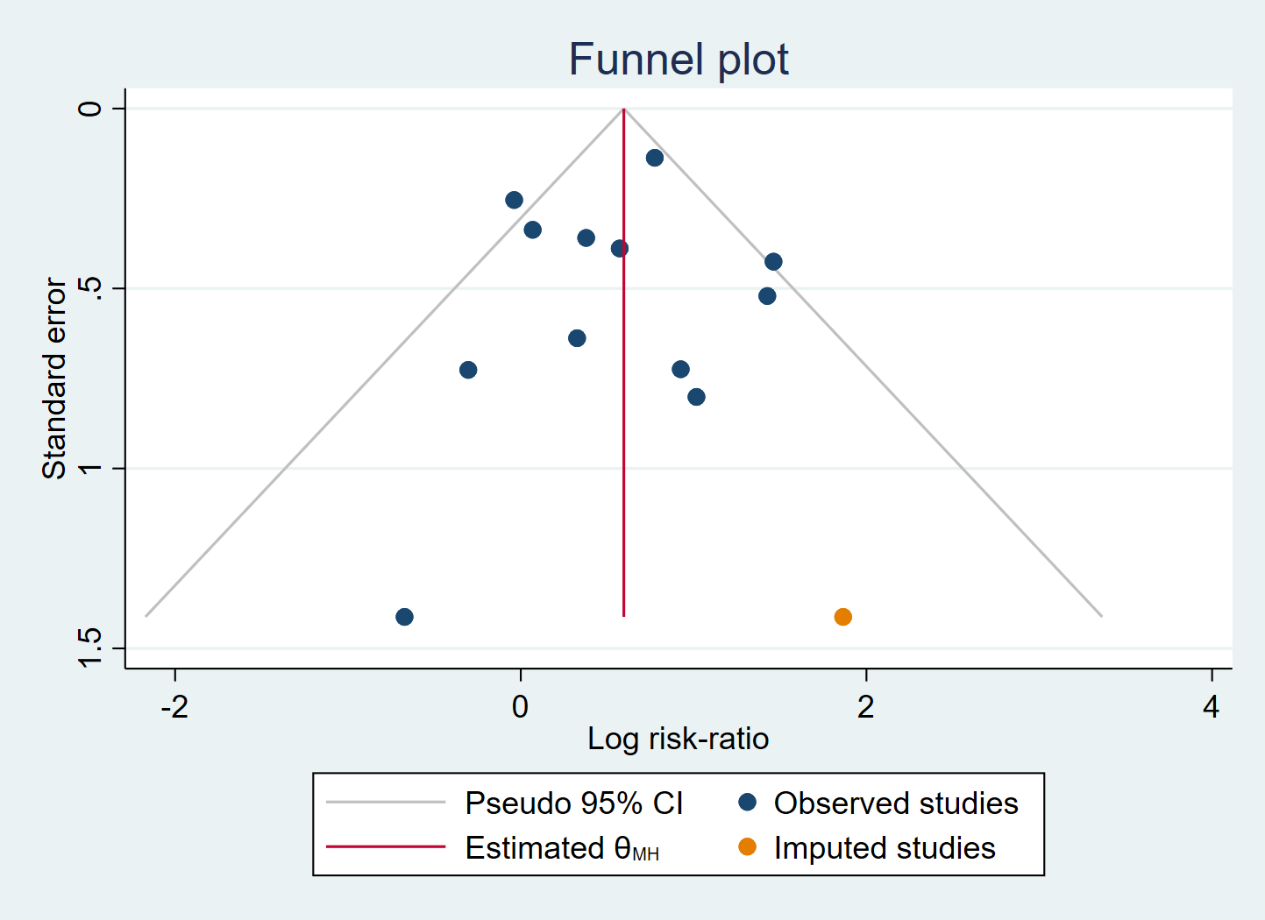
**

**Figure S43**

Funnel plot with pseudo 95% CIs. Publication bias in studies on the associations of hypertension (12 studies) with cardiotoxicity of doxorubicin. OR, odds ratio


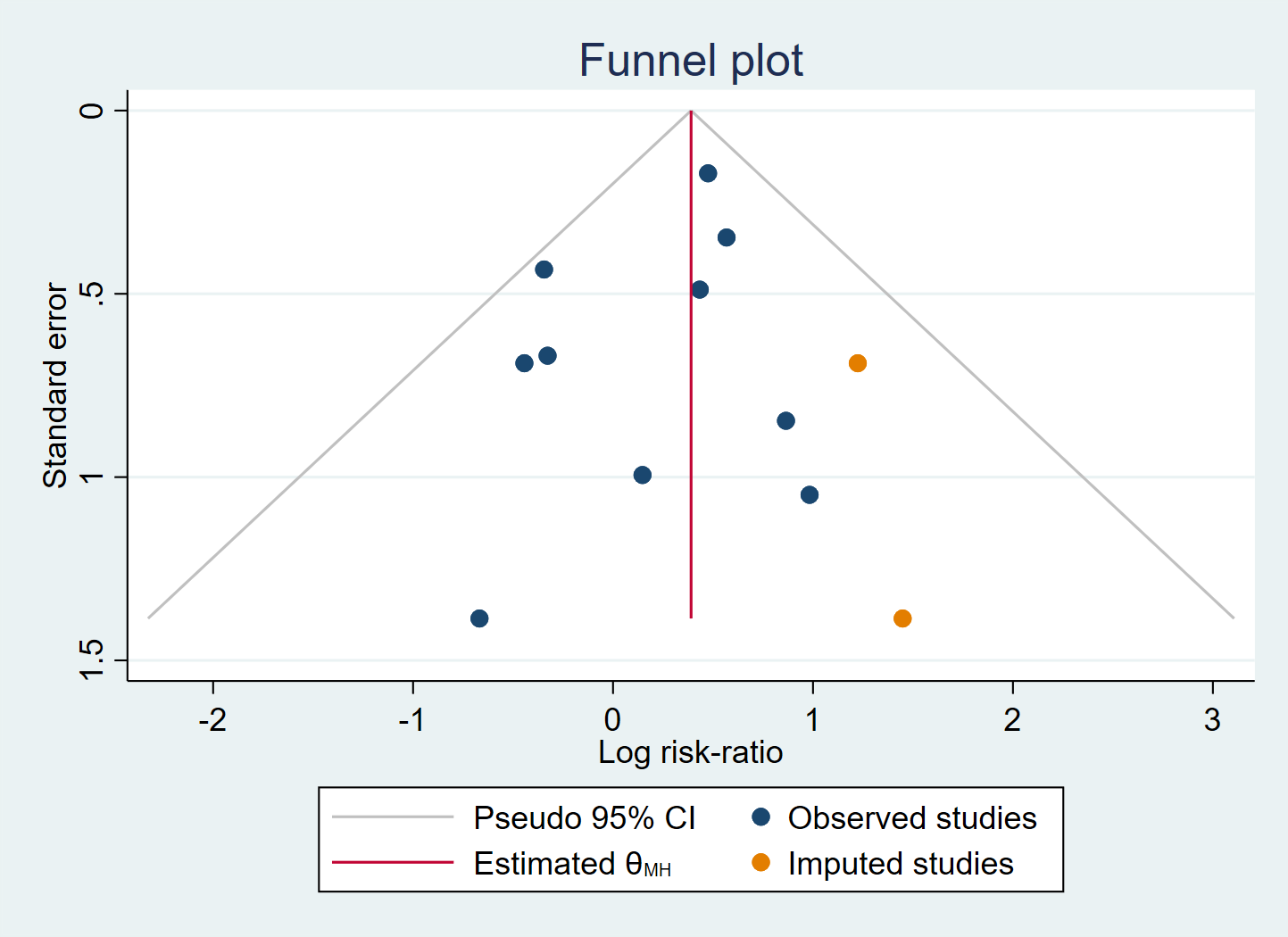


**Figure S44**

Funnel plot with pseudo 95% CIs. Publication bias in studies on the associations of diabetes (10 studies) with cardiotoxicity of doxorubicin. OR, odds ratio

Literature search strategy

### PUBMED

("Breast Neoplasms"[Title/Abstract] OR "cancer mammary"[Title/Abstract] OR "Malignant Neoplasm of Breast"[Title/Abstract] OR "cancers mammary"[Title/Abstract] OR "Breast Malignant Neoplasms"[Title/Abstract] OR "Mammary Cancer"[Title/Abstract] OR "Breast Malignant Tumors"[Title/Abstract] OR "cancer breast"[Title/Abstract] OR "Breast Malignant Tumor"[Title/Abstract] OR "Cancer of the Breast"[Title/Abstract] OR "Breast Malignant Neoplasm"[Title/Abstract] OR "Malignant Tumor of Breast"[Title/Abstract] OR "Mammary Cancers"[Title/Abstract] OR "Breast Cancer"[Title/Abstract] OR "Cancer of Breast"[Title/Abstract] OR "neoplasms breast"[Title/Abstract] OR "Breast Tumor"[Title/Abstract] OR "Breast Tumors"[Title/Abstract] OR "tumors breast"[Title/Abstract] OR "Breast Neoplasm"[Title/Abstract] OR "neoplasm breast"[Title/Abstract] OR "tumor breast"[Title/Abstract] OR "mammary carcinoma human"[Title/Abstract] OR "Human Mammary Carcinomas"[Title/Abstract] OR "Human Mammary Carcinoma"[Title/Abstract] OR "Breast Carcinomas"[Title/Abstract] OR "carcinoma breast"[Title/Abstract] OR "carcinomas breast"[Title/Abstract] OR "Breast Carcinoma"[Title/Abstract] OR "Human Mammary Neoplasms"[Title/Abstract] OR "Breast Neoplasms"[MeSH Terms]) AND ("Anthracyclines"[Title/Abstract] OR "Doxorubicin"[Title/Abstract] OR "Aclarubicin"[Title/Abstract] OR "Daunorubicin"[Title/Abstract] OR "Carubicin"[Title/Abstract] OR "Idarubicin"[Title/Abstract] OR "Nogalamycin"[Title/Abstract] OR "Plicamycin"[Title/Abstract] OR "Epirubicin"[Title/Abstract] OR "Menogaril"[Title/Abstract] OR ("Anthracyclines"[MeSH Terms] OR "Doxorubicin"[MeSH Terms] OR "Aclarubicin"[MeSH Terms] OR "Daunorubicin"[MeSH Terms] OR "Carubicin"[MeSH Terms] OR "Idarubicin"[MeSH Terms] OR "Nogalamycin"[MeSH Terms] OR "Plicamycin"[MeSH Terms] OR "Epirubicin"[MeSH Terms] OR "Menogaril"[MeSH Terms]) OR ("zavedos"[Title/Abstract] OR "tut 7"[Title/Abstract] OR "TUT7"[Title/Abstract] OR "tut 7"[Title/Abstract] OR "trixilem"[Title/Abstract] OR "Tomosar"[Title/Abstract] OR "tlc d 99"[Title/Abstract] OR "Rubomycin"[Title/Abstract] OR "Rubidomycin"[Title/Abstract] OR "Rubex"[Title/Abstract] OR "Rubeomycin"[Title/Abstract] OR "Plicamycin"[Title/Abstract] OR "Pharmorubicin"[Title/Abstract] OR "NSC82151"[Title/Abstract] OR "NSC269148"[Title/Abstract] OR "NSC256439"[Title/Abstract] OR "nsc24559"[Title/Abstract] OR "NSC208734"[Title/Abstract] OR "NSC180024"[Title/Abstract] OR "nsc123127"[Title/Abstract] OR "NSC 82151"[Title/Abstract] OR "NSC 269148"[Title/Abstract] OR "NSC 256942"[Title/Abstract] OR "NSC 256439"[Title/Abstract] OR "nsc 24559"[Title/Abstract] OR "NSC 208734"[Title/Abstract] OR "NSC 180024"[Title/Abstract] OR "nsc 123127"[Title/Abstract] OR "nogalomycin"[Title/Abstract] OR "Myocet"[Title/Abstract] OR "mitromycin*"[Title/Abstract] OR "mitramycin*"[Title/Abstract] OR "mithromycin*"[Title/Abstract] OR "Mithramycin"[Title/Abstract] OR "mithracin"[Title/Abstract] OR "methramycin"[Title/Abstract] OR "Menogarol"[Title/Abstract] OR "Menogaril"[Title/Abstract] OR "mcc465"[Title/Abstract] OR "mcc 465"[Title/Abstract] OR "lipodox"[Title/Abstract] OR "Karminomycin"[Title/Abstract] OR "IMI30"[Title/Abstract] OR "IMI28"[Title/Abstract] OR "idarubicin*"[Title/Abstract] OR "idamycin"[Title/Abstract] OR "farmorubicin*"[Title/Abstract] OR "evacet"[Title/Abstract] OR "epirubicin*"[Title/Abstract] OR "epiham"[Title/Abstract] OR "epifil"[Title/Abstract] OR "epidx"[Title/Abstract] OR "epidoxorubicin"[Title/Abstract] OR "epi cell"[Title/Abstract] OR "EPIcell"[Title/Abstract] OR "epi cell"[Title/Abstract] OR "Ellence"[Title/Abstract] OR "duanorubicin"[Title/Abstract] OR "duanomycin"[Title/Abstract] OR "doxorubin"[Title/Abstract] OR "doxorubicin*"[Title/Abstract] OR "Doxorubicin"[Title/Abstract] OR "doxo cell"[Title/Abstract] OR "doxo cell"[Title/Abstract] OR "doxil"[Title/Abstract] OR "dox sl"[Title/Abstract] OR "dexorubicin"[Title/Abstract] OR "daunoxome"[Title/Abstract] OR "daunorubidomycin"[Title/Abstract] OR "daunorubicine*"[Title/Abstract] OR "Daunorubicin"[Title/Abstract] OR "daunomycin*"[Title/Abstract] OR "Daunomycin"[Title/Abstract] OR "daunoblastin*"[Title/Abstract] OR "dauno rubidomycin*"[Title/Abstract] OR "daunarubicin"[Title/Abstract] OR "cerubidin*"[Title/Abstract] OR "Carubicin"[Title/Abstract] OR "Carminomycin"[Title/Abstract] OR "Carminomicin"[Title/Abstract] OR "caelyx/doxil"[Title/Abstract] OR "caelyx"[Title/Abstract] OR "caelix"[Title/Abstract] OR "Aureolic Acid"[Title/Abstract] OR "anthracyclin*"[Title/Abstract] OR "adrim"[Title/Abstract] OR "adriblastina"[Title/Abstract] OR "adriblastin*"[Title/Abstract] OR "adriamycin*"[Title/Abstract] OR "Adriamycin"[Title/Abstract] OR "adriamicin*"[Title/Abstract] OR "adriacin"[Title/Abstract] OR "adriablastin*"[Title/Abstract] OR "Aclarubicin"[Title/Abstract] OR "Aclaplastin"[Title/Abstract] OR "Aclacinomycin A"[Title/Abstract] OR "methylnogarol"[Title/Abstract] OR "7 omen"[Title/Abstract] OR "DXR"[Title/Abstract])) AND ("Cardiotoxicity"[Title/Abstract] OR "Cardiotoxicities"[Title/Abstract] OR "Cardiac Toxicity"[Title/Abstract] OR "Cardiac Toxicities"[Title/Abstract] OR "toxicity cardiac"[Title/Abstract] OR "cardio toxicity"[Title/Abstract] OR "cardiotoxic effect"[Title/Abstract] OR "cardiotoxicology"[Title/Abstract] OR "heart toxicity"[Title/Abstract] OR "heart failure systolic"[Title/Abstract] OR "Systolic Heart Failure"[Title/Abstract] OR "heart failure reduced ejection fraction"[Title/Abstract] OR ("Cardiotoxicity"[MeSH Terms] OR "heart failure, systolic"[MeSH Terms]))

### EMBASE

Embase

Session Results

.......................................................

No. Query Results Results Date

#12. #11 AND 'Article'/it 2,093 5 Jan 2022

#11. #7 AND #10 5,370 5 Jan 2022

#10. #8 OR #9 78,560 5 Jan 2022

#9. 'cardiotoxicity'/exp OR 'systolic heart 61,863 5 Jan 2022

failure'/exp

#8. (cardiotoxicity:ti,ab,kw OR 23,758 5 Jan 2022

'cardiotoxicities':ti,ab,kw OR 'cardiac

toxicity':ti,ab,kw OR 'cardiac

toxicities':ti,ab,kw OR 'toxicity,

cardiac':ti,ab,kw OR 'cardio toxicity':ti,ab,kw

OR 'cardiotoxic effect':ti,ab,kw OR

'cardiotoxicology':ti,ab,kw OR 'heart

toxicity':ti,ab,kw OR 'toxicity, heart':ti,ab,kw

OR 'heart failure, systolic':ti,ab,kw OR 'heart

failures, systolic':ti,ab,kw OR 'systolic heart

failures':ti,ab,kw OR 'systolic heart

failure':ti,ab,kw OR heart) AND failure, AND

reduced AND ejection AND fraction:ti,ab,kw

#7. #3 AND #6 51,651 5 Jan 2022

#6. #4 OR #5 617,103 5 Jan 2022

#5. (breast AND neoplasms:ti,ab,kw OR 'breast 39,684 5 Jan 2022

neoplasm':ti,ab,kw OR 'neoplasm, breast':ti,ab,kw

OR 'breast tumors':ti,ab,kw OR 'breast

tumor':ti,ab,kw OR 'tumor, breast':ti,ab,kw OR

'tumors, breast':ti,ab,kw OR 'neoplasms,

breast':ti,ab,kw OR 'breast cancer':ti,ab,kw OR

'cancer, breast':ti,ab,kw OR 'mammary

cancer':ti,ab,kw OR 'cancer, mammary':ti,ab,kw OR

'cancers, mammary':ti,ab,kw OR 'mammary

cancers':ti,ab,kw OR 'malignant neoplasm of

breast':ti,ab,kw OR 'breast malignant

neoplasm':ti,ab,kw OR 'breast malignant

neoplasms':ti,ab,kw OR 'malignant tumor of

breast':ti,ab,kw OR 'breast malignant

tumor':ti,ab,kw OR 'breast malignant

tumors':ti,ab,kw OR 'cancer of breast':ti,ab,kw

OR 'cancer of the breast':ti,ab,kw OR 'mammary

carcinoma, human':ti,ab,kw OR 'carcinoma, human

mammary':ti,ab,kw OR 'carcinomas, human

mammary':ti,ab,kw OR 'human mammary

carcinomas':ti,ab,kw OR 'mammary carcinomas,

human':ti,ab,kw OR 'human mammary

carcinoma':ti,ab,kw OR 'mammary neoplasms,

human':ti,ab,kw OR 'human mammary

neoplasm':ti,ab,kw OR 'human mammary

neoplasms':ti,ab,kw OR 'neoplasm, human

mammary':ti,ab,kw OR 'neoplasms, human

mammary':ti,ab,kw OR 'mammary neoplasm,

human':ti,ab,kw OR 'breast carcinoma':ti,ab,kw OR

'breast carcinomas':ti,ab,kw OR 'carcinoma,

breast':ti,ab,kw OR 'carcinomas, breast':ti,ab,kw

OR 'breasttumor':ti,ab,kw OR 'breastgland

tumor':ti,ab,kw OR 'breast gland tumour':ti,ab,kw

OR 'breast mass':ti,ab,kw OR 'breast neoplasms,

male':ti,ab,kw OR 'breast tumour':ti,ab,kw OR

'female breast neoplasm':ti,ab,kw OR 'female

breast tumor':ti,ab,kw OR 'female breast

tumour':ti,ab,kw OR 'male breast

neoplasms':ti,ab,kw OR 'mamma tumor':ti,ab,kw OR

'mamma tumour':ti,ab,kw OR 'mammary gland

tumor':ti,ab,kw OR 'mammary gland

tumour':ti,ab,kw OR 'mammary neoplasms':ti,ab,kw

OR 'mammary tumor':ti,ab,kw OR 'mammary tumor

cell':ti,ab,kw OR 'mammary tumour':ti,ab,kw OR

'mammary tumour cell':ti,ab,kw OR 'unilateral

breast neoplasms':ti,ab,kw OR

'breastneoplasms':ti,ab,kw OR

'cancer,mammary':ti,ab,kw OR

'neoplasms,breast':ti,ab,kw OR 'carcinomas,human

mammary':ti,ab,kw OR 'carcinoma,human

mammary':ti,ab,kw OR 'carcinoma,breast':ti,ab,kw

OR 'carcinomas,breast':ti,ab,kw OR

neoplasms,human) AND mammary:ti,ab,kw

#4. 'breast tumor'/exp 610,221 5 Jan 2022

#3. #1 OR #2 276,961 5 Jan 2022

#2. 'anthracycline'/exp OR 'doxorubicin'/exp OR 263,124 5 Jan 2022

'aclarubicin'/exp OR 'daunorubicin'/exp OR

'carubicin'/exp OR 'idarubicin'/exp OR

'nogalamycin'/exp OR 'mithramycin'/exp OR

'epirubicin'/exp OR 'menogaril'/exp

#1. zavedos:ti,ab,kw OR 'urokit doxo-cell':ti,ab,kw 124,621 5 Jan 2022

OR 'urokit doxo cell':ti,ab,kw OR

'u15167':ti,ab,kw OR 'u 52047':ti,ab,kw OR

'tut-7':ti,ab,kw OR 'tut7':ti,ab,kw OR 'tut

7':ti,ab,kw OR 'trixilem':ti,ab,kw OR

'tomosar':ti,ab,kw OR 'tlc d 99':ti,ab,kw OR

'sarcodoxome':ti,ab,kw OR 'rubomycine c':ti,ab,kw

OR 'rubomycin':ti,ab,kw OR 'rubilem':ti,ab,kw OR

'rubidox':ti,ab,kw OR 'rubidomycin':ti,ab,kw OR

'rubidiomycin*':ti,ab,kw OR 'rubex':ti,ab,kw OR

'rubeomycin':ti,ab,kw OR 'rp25253':ti,ab,kw OR

'rp13057':ti,ab,kw OR 'ribodoxo':ti,ab,kw OR

'resmycin':ti,ab,kw OR 'rastocin':ti,ab,kw OR

'plicamycin':ti,ab,kw OR 'pidorubicin':ti,ab,kw

OR 'pharmorubucin rd':ti,ab,kw OR

'pharmorubicine':ti,ab,kw OR

'pharmorubicin':ti,ab,kw OR 'pa144':ti,ab,kw OR

'onkodox':ti,ab,kw OR 'nsc82151':ti,ab,kw OR

'nsc70845':ti,ab,kw OR 'nsc269148':ti,ab,kw OR

'nsc256942':ti,ab,kw OR 'nsc256439':ti,ab,kw OR

'nsc24559':ti,ab,kw OR 'nsc208734':ti,ab,kw OR

'nsc180024':ti,ab,kw OR 'nsc123127':ti,ab,kw OR

'nsc 82151':ti,ab,kw OR 'nsc 269148':ti,ab,kw OR

'nsc 256942':ti,ab,kw OR 'nsc 256439':ti,ab,kw OR

'nsc 24559':ti,ab,kw OR 'nsc 208734':ti,ab,kw OR

### COCHRANE

Search Name: 乳腺癌心脏毒性COCHRANE 检索

Last Saved: 03/01/2022 11:01:51

Comment: meta

ID Search

#1 Cardiac Toxicities OR Systolic Heart Failures

#2 (Cardiotoxicity):ab,ti,kw OR (Cardiotoxicities):ab,ti,kw OR (Cardiac Toxicity):ab,ti,kw OR (Cardiac Toxicities):ab,ti,kw OR (Toxicity, Cardiac):ab,ti,kw OR (cardio toxicity):ab,ti,kw OR (cardiotoxic effect):ab,ti,kw OR (cardiotoxicology):ab,ti,kw OR (heart toxicity):ab,ti,kw OR (toxicity, heart):ab,ti,kw OR (Heart Failure, Systolic):ab,ti,kw OR (Heart Failures, Systolic):ab,ti,kw OR (Systolic Heart Failures):ab,ti,kw OR (Systolic Heart Failure):ab,ti,kw OR (Heart Failure, Reduced Ejection Fraction):ab,ti,kw

#3 #1 OR #2

#4 ”Anthracyclines”OR”Doxorubicin”OR”Aclarubicin”OR”Daunorubicin”OR”Carubicin”OR”idarubicin”OR”Nogalamycin”OR”Plicamycin”OR”Epirubicin”OR”Menogaril”

#5 (zavedos):ab,ti,kw OR (Urokit Doxo-cell):ab,ti,kw OR (Urokit Doxo cell):ab,ti,kw OR (U15167):ab,ti,kw OR (u 52047):ab,ti,kw OR (TUT-7):ab,ti,kw OR (TUT7):ab,ti,kw OR (TUT 7):ab,ti,kw OR (trixilem):ab,ti,kw OR (Tomosar):ab,ti,kw OR (tlc d 99):ab,ti,kw OR (sarcodoxome):ab,ti,kw OR (rubomycine c):ab,ti,kw OR (Rubomycin):ab,ti,kw OR (rubilem):ab,ti,kw OR (rubidox):ab,ti,kw OR (Rubidomycin):ab,ti,kw OR (rubidiomycin*):ab,ti,kw OR (Rubex):ab,ti,kw OR (Rubeomycin):ab,ti,kw OR (rp25253):ab,ti,kw OR (rp13057):ab,ti,kw OR (Ribodoxo):ab,ti,kw OR (resmycin):ab,ti,kw OR (rastocin):ab,ti,kw OR (Plicamycin):ab,ti,kw OR (pidorubicin):ab,ti,kw OR (pharmorubucin rd):ab,ti,kw OR (pharmorubicine):ab,ti,kw OR (Pharmorubicin):ab,ti,kw OR (pa144):ab,ti,kw OR (Onkodox):ab,ti,kw OR (NSC82151):ab,ti,kw OR (nsc70845):ab,ti,kw OR (NSC269148):ab,ti,kw OR (NSC256942):ab,ti,kw OR (NSC256439):ab,ti,kw OR (nsc24559):ab,ti,kw OR (NSC208734):ab,ti,kw OR (NSC180024):ab,ti,kw OR (nsc123127):ab,ti,kw OR (NSC 82151):ab,ti,kw OR (NSC 269148):ab,ti,kw OR (NSC 256942):ab,ti,kw OR (NSC 256439):ab,ti,kw OR (nsc 24559):ab,ti,kw OR (NSC 208734):ab,ti,kw OR (NSC 180024):ab,ti,kw OR (NSC 180,024):ab,ti,kw OR (nsc 123127):ab,ti,kw OR (nogarol, 7 o methyl):ab,ti,kw OR (nogalomycin):ab,ti,kw OR (ndc00824155):ab,ti,kw OR (mytramycin):ab,ti,kw OR (Myocet):ab,ti,kw OR (mitromycin*):ab,ti,kw OR (Mitramycin*):ab,ti,kw OR (mithromycin*):ab,ti,kw OR (Mithramycin):ab,ti,kw OR (mithracyne):ab,ti,kw OR (mithracin):ab,ti,kw OR (mithiamycin):ab,ti,kw OR (methramycin):ab,ti,kw OR (methracin):ab,ti,kw OR (Menogarol):ab,ti,kw OR (Menogaril):ab,ti,kw OR (mcc465):ab,ti,kw OR (mcc 465):ab,ti,kw OR (maxidauno):ab,ti,kw OR (MA144A1):ab,ti,kw OR (lipodox):ab,ti,kw OR (Karminomycin):ab,ti,kw OR (Karminomicin):ab,ti,kw OR (jaclacin):ab,ti,kw OR (IMI30):ab,ti,kw OR (IMI28):ab,ti,kw OR (ifadox):ab,ti,kw OR (Idarubicin*):ab,ti,kw OR (idaralem):ab,ti,kw OR (idamycin):ab,ti,kw OR (fi6339):ab,ti,kw OR (fi106):ab,ti,kw OR (Farmorubicin*):ab,ti,kw OR (farmorrubicina rtu):ab,ti,kw OR (Farmiblastina):ab,ti,kw OR (evacet):ab,ti,kw OR (Epirubicin*):ab,ti,kw OR (Epilem):ab,ti,kw OR (epiham):ab,ti,kw OR (epifil):ab,ti,kw OR (epidx):ab,ti,kw OR (epidoxorubicin):ab,ti,kw OR (epidoxo):ab,ti,kw OR (EPI-cell):ab,ti,kw OR (EPIcell):ab,ti,kw OR (EPI cell):ab,ti,kw OR (Ellence):ab,ti,kw OR (duanorubicin):ab,ti,kw OR (duanomycin):ab,ti,kw OR (Doxotec):ab,ti,kw OR (doxorubin):ab,ti,kw OR (Doxorubicin*):ab,ti,kw OR (Doxorubicin):ab,ti,kw OR (doxor lyo):ab,ti,kw OR (doxolipad):ab,ti,kw OR (Doxolem):ab,ti,kw OR (DOXO-cell):ab,ti,kw OR (DOXO cell):ab,ti,kw OR (doxil):ab,ti,kw OR (dox sl):ab,ti,kw OR (dexorubicin):ab,ti,kw OR (daurorubicin):ab,ti,kw OR (daunoxome):ab,ti,kw OR (daunorubimycin):ab,ti,kw OR (Dauno-Rubidomycine):ab,ti,kw OR (daunorubidomycin):ab,ti,kw OR (daunorubicine*):ab,ti,kw OR (Daunorubicin):ab,ti,kw OR (daunorrubicina):ab,ti,kw OR (daunomycin*):ab,ti,kw OR (daunoextra):ab,ti,kw OR (Daunoblastin*):ab,ti,kw OR (daunobin):ab,ti,kw OR (dauno rubidomycin*):ab,ti,kw OR (daunarubicin):ab,ti,kw OR (daunamycin):ab,ti,kw OR (dannomycin):ab,ti,kw OR (damycin):ab,ti,kw OR (cerubidin*):ab,ti,kw OR (Carubicin):ab,ti,kw OR (carmynomycin):ab,ti,kw OR (Carminomycin):ab,ti,kw OR (carminomitsin):ab,ti,kw OR (Carminomicin):ab,ti,kw OR (carcinocin):ab,ti,kw OR (caelyx):ab,ti,kw OR (caelix):ab,ti,kw OR (binarin):ab,ti,kw OR (AureolicAcid):ab,ti,kw OR (Aureolic Acid):ab,ti,kw OR (aurelic acid):ab,ti,kw OR (antibiotic ma 144a1):ab,ti,kw OR (anthracyclin*):ab,ti,kw OR (amminac):ab,ti,kw OR (adrubicin):ab,ti,kw OR (Adrimedac):ab,ti,kw OR (adrim):ab,ti,kw OR (adriblastina):ab,ti,kw OR (Adriblastin*):ab,ti,kw OR (adriamycin*):ab,ti,kw OR (Adriamycin):ab,ti,kw OR (adriamicin*):ab,ti,kw OR (adriacin):ab,ti,kw OR (Adriablastin*):ab,ti,kw OR (Aclarubicin):ab,ti,kw OR (Aclaplastin):ab,ti,kw OR (aclacinon):ab,ti,kw OR (Aclacinomycin A):ab,ti,kw OR (Aclacin):ab,ti,kw OR (a2371):ab,ti,kw OR (a.d.mycin):ab,ti,kw OR (methylnogarol):ab,ti,kw OR (7 omen):ab,ti,kw OR (DXR):ab,ti,kw

#6 #4 OR #5

#7

#8 Breast Neoplasms

#9 (Cancer, Mammary):ab,ti,kw OR ( Malignant Neoplasm of Breast):ab,ti,kw OR ( Cancers, Mammary):ab,ti,kw OR ( Breast Malignant Neoplasms):ab,ti,kw OR ( Mammary Cancer):ab,ti,kw OR ( Breast Malignant Tumors):ab,ti,kw OR ( Cancer, Breast):ab,ti,kw OR ( Breast Malignant Tumor):ab,ti,kw OR ( Cancer of the Breast):ab,ti,kw OR ( Breast Malignant Neoplasm):ab,ti,kw OR ( Malignant Tumor of Breast):ab,ti,kw OR ( Mammary Cancers):ab,ti,kw OR ( Breast Cancer):ab,ti,kw OR ( Cancer of Breast):ab,ti,kw OR (Neoplasms, Breast):ab,ti,kw OR ( Breast Tumor):ab,ti,kw OR ( Breast Tumors):ab,ti,kw OR ( Tumors, Breast):ab,ti,kw OR ( Breast Neoplasm):ab,ti,kw OR ( Neoplasm, Breast):ab,ti,kw OR ( Tumor, Breast):ab,ti,kw OR (Carcinomas, Human Mammary):ab,ti,kw OR ( Mammary Carcinoma, Human):ab,ti,kw OR ( Human Mammary Carcinomas):ab,ti,kw OR ( Mammary Carcinomas, Human):ab,ti,kw OR ( Human Mammary Carcinoma):ab,ti,kw OR (Carcinoma, Human Mammary):ab,ti,kw OR ( Breast Carcinomas):ab,ti,kw OR (Carcinoma, Breast):ab,ti,kw OR (Carcinomas, Breast):ab,ti,kw OR ( Breast Carcinoma):ab,ti,kw OR ( Mammary Neoplasm, Human):ab,ti,kw OR (Neoplasms, Human Mammary):ab,ti,kw OR ( Mammary Neoplasms, Human):ab,ti,kw OR ( Neoplasm, Human Mammary):ab,ti,kw OR ( Human Mammary Neoplasm):ab,ti,kw OR ( Human Mammary Neoplasms):ab,ti,kw

#10 #8 OR #9

#11 #6 AND #10

#12 #11 AND #3

## Literature quality evaluation

### case control study

| Author, year | selection | | | | Comparability | Exposure | | |
| --- | --- | --- | --- | --- | --- | --- | --- | --- |
|  | #1 | #2 | #3 | #4 | #1 | #1 | #2 | #3 |
| reinbolt 2016 | a | a | b | a | a | d | a | b |
| He 2017 | a | b | b | a | a | a | a | b |
| Todorova2020 | a | a | b | a | a | a | a | b |
| Egashira 2021 | a | a | b | a | a | a | a | b |

Selection

1) Is the case definition adequate?

a) yes, with independent validation Ø

b) yes, eg record linkage or based on self reports

c) no description

2) Representativeness of the cases

a) consecutive or obviously representative series of cases Ø

b) potential for selection biases or not stated

3) Selection of Controls

a) community controls Ø

b) hospital controls

c) no description

4) Definition of Controls

a) no history of disease (endpoint) Ø

b) no description of source

Comparability

1) Comparability of cases and controls on the basis of the design or analysis

a) study controls for _______________ (Select the most important factor.) Ø

b) study controls for any additional factor Ø (This criteria could be modified to indicate specific

control for a second important factor.)

Exposure

1) Ascertainment of exposure

a) secure record (eg surgical records) Ø

b) structured interview where blind to case/control status Ø

c) interview not blinded to case/control status

d) written self report or medical record only

e) no description

2) Same method of ascertainment for cases and controls

a) yes Ø

b) no

3) Non-Response rate

a) same rate for both groups Ø

b) non respondents described

c) rate different and no designation

### cross-sectional study

| mina2015 | HRQ11 |
| --- | --- |
| buzdar-1985 | HRQ8 |
| pinder-2007 | HRQ8 |
| chung-2013 | HRQ9 |
| caram-2015 | HRQ9 |
| mina-2015 | HRQ10 |
| kim-2017 | HRQ9 |
| cho-2020 | HRQ10 |
| fogarassy-2020 | HRQ6 |

1. Was the spectrum of patients representative of the patients who will receive the test in practice?

2. Were selection criteria clearly described?

3. Is the reference standard likely to correctly classify the target condition?

4. Is the time period between reference standard and index test short enough to be reasonably sure that the target condition did not change between the two tests?

5. Did the whole sample or a random selection of the sample, receive verification using a reference standard of diagnosis?

6. Did patients receive the same reference standard regardless of the index test result?

7. Was the reference standard independent of the index test (i.e. the index test did not form part of the reference standard)?

8a. Was the execution of the index test described in sufficient detail to permit replication of the test?

8b. Was the execution of the reference standard described in sufficient detail to permit its replication?

9a. Were the index test results interpreted without knowledge of the results of the reference standard?

9b. Were the reference standard results interpreted without knowledge of the results of the index test?

10. Were the same clinical data available when test results were interpreted as would be available when the test is used in practice?

11. Were uninterpretable/ intermediate test results reported?

12. Were withdrawals from the study explained?

### randomized controlled trial


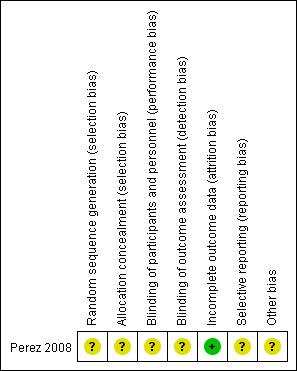


**Figure S34**

Literature quality evaluation of randomized controlled studies

### cohort studies

| gennari 1999 | NOS7 |
| --- | --- |
| sawaya 2011 | NOS7 |
| Vivenza 2013 | NOS7 |
| serrano2015 | NOS8 |
| kotwinski 2016 | NOS7 |
| Matos2016 | NOS7 |
| Pearson2017 | NOS7 |
| Stachowiak2017 | NOS7 |
| S. P. Wu, M-2017 | NOS8 |
| El-Sherbeny 2019 | NOS6 |
| li-2019 | NOS7 |
| ruger2020 | NOS7 |
| L TAN-2020 | NOS7 |
| Vaitiekus2020 | NOS7 |
| Feng 2021 | NOS7 |
| Houbois 2021 | NOS6 |
| santos 2020 | NOS7 |
| ganz 2017 | NOS6 |
| Aitelhaj2013 | NOS8 |

NEWCASTLE - OTTAWA QUALITY ASSESSMENT SCALE

COHORT STUDIES

Note: A study can be awarded a maximum of one star for each numbered item within the Selection and

Outcome categories. A maximum of two stars can be given for Comparability

Selection

1) Representativeness of the exposed cohort

a) truly representative of the average _______________ (describe) in the community Ø

b) somewhat representative of the average ______________ in the community Ø

c) selected group of users eg nurses, volunteers

d) no description of the derivation of the cohort

2) Selection of the non exposed cohort

a) drawn from the same community as the exposed cohort Ø

b) drawn from a different source

c) no description of the derivation of the non exposed cohort

3) Ascertainment of exposure

a) secure record (eg surgical records) Ø

b) structured interview Ø

c) written self report

d) no description

4) Demonstration that outcome of interest was not present at start of study

a) yes Ø

b) no

Comparability

1) Comparability of cohorts on the basis of the design or analysis

a) study controls for _____________ (select the most important factor) Ø

b) study controls for any additional factor Ø (This criteria could be modified to indicate specific

control for a second important factor.)

Outcome

1) Assessment of outcome

a) independent blind assessment Ø

b) record linkage Ø

c) self report

d) no description

2) Was follow-up long enough for outcomes to occur

a) yes (select an adequate follow up period for outcome of interest) Ø

b) no

3) Adequacy of follow up of cohorts

a) complete follow up - all subjects accounted for Ø

b) subjects lost to follow up unlikely to introduce bias - small number lost - > ____ % (select an

adequate %) follow up, or description provided of those lost) Ø

c) follow up rate < ____% (select an adequate %) and no description of those lost

d) no statemen

## Literature inclusion and exclusion

### Literature inclusion criteria

| Participantse | Patients with breast cancer who are receiving anthracyclines |
| --- | --- |
| Exposure | Patients with cardiovascular risk factors |
| Control | Patients without cardiovascular risk factors |
| Outcomes | All cardiac events |
| Study design | Case control，cohort study，randomized controlled trial |

### Literature exclusion

| Excluded studies | Reason |
| --- | --- |
| Ali-1983(1) | No related data is included |
| Robinson-1983(2) | No related data is included |
| Allegra-1985(3) | No related data is included |
| Mohamed-1985(4) | No related data is included |
| Wander-1987(5) | No related data is included |
| Bezwoda-1990(6) | No related data is included |
| Ringenberg-1990(7) | No related data is included |
| Graaf-1997(8) | No related data is included |
| Michelotti-2000(9) | No related data is included |
| Mitani-2003(10) | No related data is included |
| Rohde-2005(11) | patients are not all with breat cancer |
| Sukel-2008(12) | No related data is included |
| Abdurrahman-2010(13) | No related data is included |
| Appel-2010(14) | No related data is included |
| Ho-2010(15) | No related data is included |
| Rocca-2010(16) | No related data is included |
| Cochet-2011(17) | patient are not all treated with anthracycline |
| Dogan-2012(18) | No related data is included |
| Piotrowski-2012(19) | patient are not all treated with anthracycline |
| Yood-2012(20) | No related data is included |
| Piotrowski-2013(21) | patient are not all treated with anthracycline |
| Tzonevska-2014(22) | No related data is included |
| Wang-2014(23) | No related data is included |
| Bulten-2015(24) | No related data is included |
| Cao-2015(25) | No related data is included |
| Yu-2015(26) | patient are not all treated with anthracycline |
| Advani-2016(27) | patients are not all with breat cancer |
| Helen-2016(28) | patient are not all treated with anthracycline |
| Fei-2016(29) | No related data is included |
| Gunaldi-2016(30) | patient are not all treated with anthracycline |
| murtagh-2016(31) | No related data is included |
| Narayan-2016(32) | No related data is included |
| Yoon-2016(33) | patient are not all treated with anthracycline |
| Yu-2016(34) | No related data is included |
| Geeta-2017(35) | No related data is included |
| Boyd-2017(36) | No related data is included |
| khan-2017(37) | The definition of cardiotoxicity are unclear |
| law-2017(38) | No related data is included |
| Mi-2017(39) | patient are not all treated with anthracycline |
| saiki-2017(40) | patient are not all treated with anthracycline |
| Skubitz-2017(41) | No related data is included |
| Tang-2017(42) | patient are not all treated with anthracycline |
| Toufan-2017(43) | No related data is included |
| Abdel-2018(44) | No related data is included |
| Maria-2018(45) | No related data is included |
| freres-2018(46) | No related data is included |
| Jordan-2018(47) | patients are not all with breat cancer |
| Wittayanukorn-2018(48) | patient are not all treated with anthracycline |
| yu-2018(49) | No related data is included |
| Maysa-2019(50) | No related data is included |
| Zainab-2019(51) | No related data is included |
| Antolin-2019(52) | No related data is included |
| Cancle-2019(53) | patients are not all with breat cancer |
| Clark-2019(54) | patients are not all with breat cancer |
| de barrors-2019(55) | patient are not all treated with anthracycline |
| kim-2019(56) | patient are not all treated with anthracycline |
| Klein-2019(57) | patient are not all treated with anthracycline |
| Kosalka-2019(58) | No related data is included |
| Martinello-2019(59) | No related data is included |
| Ichrak-2020(60) | No related data is included |
| Demissei-2020(61) | patient are not all treated with anthracycline |
| Eiger-2020(62) | patient are not all treated with anthracycline |
| Fang-2020(63) | No related data is included |
| Foulkes-2020(64) | No related data is included |
| Iqbal-2020(65) | No related data is included |
| Laufer-2020(66) | patients are not all with breat cancer |
| Sung-2020(67) | patient are not all treated with anthracycline |
| Upshaw-2020(68) | patient are not all treated with anthracycline |
| Yu-2020(69) | No related data is included |
| Yu-2020(70) | patient are not all treated with anthracycline |
| Husam-2021(71) | No related data is included |
| Raquel-2021(72) | patients are not all with breat cancer |
| Azhar-2021(73) | No related data is included |
| Souza-2021(74) | No related data is included |
| Douganiotis-2021(75) | No related data is included |
| Fradley-2021(76) | No related data is included |
| Haffadi-2021(77) | patient are not all treated with anthracycline |
| kim-2021(78) | patient are not all treated with anthracycline |
| Lee-2021(79) | No related data is included |
| Puckett-2021(80) | No related data is included |
| Reding-2021(81) | No related data is included |
| Simoes-2021(82) | No related data is included |
| Subramaniam-2021(83) | No related data is included |
| Sulaiman-2021(84) | No related data is included |
| Tang-2021(85) | No related data is included |
| Vaitiekus-2021(86) | No related data is included |
| Varghese-2021(87) | No related data is included |
| Vo-2021(88) | patient are not all treated with anthracycline |
| Yao-2021(89) | No related data is included |
| Yoodee-2021(90) | No related data is included |

1. Ali MK, Buzdar AU, Ewer M. Noninvasive cardiac evaluation of patients receiving adriamycin-containing adjuvant chemotherapy (FAC) for state II or III breast cancer. Journal of Surgical Oncology. 1983;23(3):212-6.

2. Robinson BA, Colls BM, Turner JG. Adriamycin cardiotoxicity monitoring by radionuclide scan. British Journal of Cancer. 1983;48(2):315-7.

3. Allegra JC, Woodcock T, Woolf S. A randomized trial comparing mitoxantrone with doxorubicin in patients with stage IV breast cancer. Investigational New Drugs. 1985;3(2):153-61.

4. Haq MM, Legha SS, Choksi J. Doxorubicin-induced congestive heart failure in adults. Cancer. 1985;56(6):1361-5.

5. Wander HE, Nagel GA, Luig H, Emrich D. Intensive short-term chemotherapy in patients with advanced breast cancer. Klin Wochenschr. 1987;65(7):317-23.

6. Bezwoda WR, Dansey R, Seymour L. High-dose 4'-epiadriamycin for treatment of breast cancer refractory to standard dose anthracycline chemotherapy: achievement of second responses. Oncology. 1990;47(1):4-8.

7. Ringenberg QS, Propert KJ, Muss HB, Weiss RB, Schilsky RL, Modeas C, et al. Clinical cardiotoxicity of esorubicin (4'-deoxydoxorubicin,DxDx): prospective studies with serial gated heart scans and reports of selected cases. A Cancer and Leukemia Group B report. Invest New Drugs. 1990;8(2):221-6.

8. De Graaf H, Dolsma WV, Willemse PHB, Van Der Graaf WTA, Sleijfer DT, De Vries EGE, et al. Cardiotoxicity from intensive chemotherapy combined with radiotherapy in breast cancer. British Journal of Cancer. 1997;76(7):943-5.

9. Michelotti A, Venturini M, Tibaldi C, Bengala C, Gallo L, Carnino F, et al. Single agent epirubicin as first line chemotherapy for metastatic breast cancer patients. Breast cancer research and treatment. 2000;59(2):133‐9.

10. Mitani I, Jain D, Joska TM, Burtness B, Zaret BL. Doxorubicin cardiotoxicity: Prevention of congestive heart failure with serial cardiac function monitoring with equilibrium radinuclide angiocardiography in the current era. Journal of Nuclear Cardiology. 2003;10(2):132-9.

11. Rohde LE, Bello-Klein A, Pereira RP, Mazzotti NG, Geib G, Weber C, et al. Superoxide dismutase activity in adriamycin-induced cardiotoxicity in humans: a potential novel tool for risk stratification. Journal of cardiac failure. 2005;11(3):220‐6.

12. Sukel MPP, Breekveldt-Postma NS, Erkens JA, van der Linden PD, Beiderbeck AB, Coebergh JWW, et al. Incidence of cardiovascular events in breast cancer patients receiving chemotherapy in clinical practice. Pharmacoepidemiology and Drug Safety. 2008;17(2):125-34.

13. Aldiab A. Cardiotoxicity with adjuvant trastuzumab use in breast cancer: A single institution»s experience. Journal of the Saudi Heart Association. 2010;22(3):133-6.

14. Appel JM, Jensen BV, Nielsen DL, Ryberg M, Zerahn B. Systolic versus diastolic cardiac function variables during epirubicin treatment for breast cancer. International Journal of Cardiovascular Imaging. 2010;26(2):217-23.

15. Ho E, Brown A, Barrett P, Morgan RB, King G, Kennedy MJ, et al. Subclinical anthracycline- and trastuzumab-induced cardiotoxicity in the long-term follow-up of asymptomatic breast cancer survivors: A speckle tracking echocardiographic study. Heart. 2010;96(9):701-7.

16. Rocca A, Maltoni R, Passardi A, Massa I, Aquilina M, Ridolfi R, et al. A phase IB dose-finding trial of liposomal doxorubicin in combination with capecitabine in patients with pretreated metastatic breast cancer. Cancer Chemotherapy and Pharmacology. 2010;65(5):871-6.

17. Cochet A, Quilichini G, Dygai-Cochet I, Touzery C, Toubeau M, Berriolo-Riedinger A, et al. Baseline diastolic dysfunction as a predictive factor of trastuzumab-mediated cardiotoxicity after adjuvant anthracycline therapy in breast cancer. Breast Cancer Research and Treatment. 2011;130(3):845-54.

18. Dogan E, Yorgun H, Petekkaya I, Ozer N, Altundag K, Ozisik Y. Evaluation of cardiac safety of lapatinib therapy for ErbB2-positive metastatic breast cancer: A single center experience. Medical Oncology. 2012;29(5):3232-9.

19. Piotrowski G, Gawor R, Stasiak A, Gawor Z, Potemski P, Banach M. Cardiac complications associated with trastuzumab in the setting of adjuvant chemotherapy for breast cancer overexpressing human epidermal growth factor receptor type 2 - A prospective study. Archives of Medical Science. 2012;8(2):227-35.

20. Yood MU, Wells KE, Alford SH, Dakki H, Beiderbeck AB, Hurria A, et al. Cardiovascular outcomes in women with advanced breast cancer exposed to chemotherapy. Pharmacoepidemiology and Drug Safety. 2012;21(8):818-27.

21. Piotrowski G, Gawor R, Bourge RC, Stasiak A, Potemski P, Gawor Z, et al. Heart remodeling induced by adjuvant trastuzumab-containing chemotherapy for breast cancer overexpressing human epidermal growth factor receptor type 2: A prospective study. Pharmacological Research. 2013;78:41-8.

22. Tzonevska A, Chakarova A, Tzvetkov K. GSPECT-CT myocardial scintigraphy plus calcium scores as screening tool for prevention of cardiac side effects in left-sided breast cancer radiotherapy. Journal of BUON. 2014;19(3):667-72.

23. Wang SY, Long JB, Hurria A, Owusu C, Steingart RM, Gross CP, et al. Cardiovascular events, early discontinuation of trastuzumab, and their impact on survival. Breast Cancer Research and Treatment. 2014;146(2):411-9.

24. Bulten BF, Verberne HJ, Bellersen L, Oyen WJG, Sabaté-Llobera A, Mavinkurve-Groothuis AMC, et al. Relationship of promising methods in the detection of anthracycline-induced cardiotoxicity in breast cancer patients. Cancer Chemotherapy and Pharmacology. 2015;76(5):957-67.

25. Cao L, Cai G, Chang C, Miao AY, Yu XL, Yang ZZ, et al. Diastolic dysfunction occurs early in HER2-positive breast cancer patients treated concurrently with radiation therapy and trastuzumab. Oncologist. 2015;20(6):605-14.

26. Yu AF, Yadav NU, Lung BY, Eaton AA, Thaler HT, Hudis CA, et al. Trastuzumab interruption and treatment-induced cardiotoxicity in early HER2-positive breast cancer. Breast Cancer Research and Treatment. 2015;149(2):489-95.

27. Advani PP, Ballman KV, Dockter TJ, Colon-Otero G, Perez EA. Long-term cardiac safety analysis of NCCTG N9831 (Alliance) adjuvant trastuzumab trial. Journal of clinical oncology. 2016;34(6):581‐7.

28. Earl HM, Vallier AL, Dunn J, Loi S, Ogburn E, McAdam K, et al. Trastuzumab-associated cardiac events in the Persephone trial. British journal of cancer. 2016;115(12):1462‐70.

29. Fei HW, Ali MT, Tan TC, Cheng KH, Salama L, Hua L, et al. Left Ventricular Global Longitudinal Strain in HER-2 + Breast Cancer Patients Treated with Anthracyclines and Trastuzumab Who Develop Cardiotoxicity Is Associated with Subsequent Recovery of Left Ventricular Ejection Fraction. Echocardiography. 2016;33(4):519-26.

30. Koelwyn GJ, Lewis NC, Ellard SL, Jones LW, Gelinas JC, Rolf JD, et al. Ventricular-Arterial Coupling in Breast Cancer Patients After Treatment With Anthracycline-Containing Adjuvant Chemotherapy. Oncologist. 2016;21(2):141-9.

31. Murtagh G, Lyons T, O'Connell E, Ballot J, Geraghty L, Fennelly D, et al. Late cardiac effects of chemotherapy in breast cancer survivors treated with adjuvant doxorubicin: 10-year follow-up. Breast Cancer Res Treat. 2016;156(3):501-6.

32. Narayan HK, French B, Khan AM, Plappert T, Hyman D, Bajulaiye A, et al. Noninvasive Measures of Ventricular-Arterial Coupling and Circumferential Strain Predict Cancer Therapeutics–Related Cardiac Dysfunction. JACC: Cardiovascular Imaging. 2016;9(10):1131-41.

33. Yoon HJ, Kim KH, Kim JY, Park HJ, Cho JY, Hong YJ, et al. Chemotherapy-induced left ventricular dysfunction in patients with breast cancer. Journal of Breast Cancer. 2016;19(4):402-9.

34. Yu AF, Manrique C, Pun S, Liu JE, Mara E, Fleisher M, et al. Cardiac Safety of Paclitaxel Plus Trastuzumab and Pertuzumab in Patients With HER2-Positive Metastatic Breast Cancer. Oncologist. 2016;21(4):418-24.

35. Neurohormonal blockade and circulating cardiovascular biomarkers during anthracycline therapy in breast cancer patients: results from the PRADA (Prevention of Cardiac Dysfunction During Adjuvant Breast Cancer Therapy) study. Journal of the american heart association. 2017;6(11).

36. Boyd A, Stoodley P, Richards D, Hui R, Harnett P, Vo K, et al. Anthracyclines induce early changes in left ventricular systolic and diastolic function: A single centre study. PLoS ONE. 2017;12(4).

37. Khan M, Siddiqui SA, Gupta MK, Seam RK, Gupta M. Normal tissue complications following hypofractionated chest wall radiotherapy in breast cancer patients and their correlation with patient, tumor, and treatment characteristics. Indian Journal of Medical and Paediatric Oncology. 2017;38(2):121-7.

38. Law W, Johnson C, Rushton M, Dent S. The framingham risk score underestimates the risk of cardiovascular events in the HER2-positive breast cancer population. Current Oncology. 2017;24(5):e348-e53.

39. Lee MH, Yee J, Kim YJ, Moon JY, Kim JH, Rhie SJ, et al. Factors for time to trastuzumab-induced cardiotoxicity in breast cancer patients. Medical Oncology. 2017;34(12).

40. Saiki H, Petersen IA, Scott CG, Bailey KR, Dunlay SM, Finley RR, et al. Risk of Heart Failure with Preserved Ejection Fraction in Older Women after Contemporary Radiotherapy for Breast Cancer. Circulation. 2017;135(15):1388-96.

41. Skubitz KM, Blaes AH, Konety SH, Francis GS. Cardiac safety profile of patients receiving high cumulative doses of pegylated-liposomal doxorubicin: use of left ventricular ejection fraction is of unproven value. Cancer Chemotherapy and Pharmacology. 2017;80(4):787-98.

42. Tang GH, Acuna SA, Sevick L, Yan AT, Brezden-Masley C. Incidence and identification of risk factors for trastuzumab-induced cardiotoxicity in breast cancer patients: an audit of a single “real-world” setting. Medical Oncology. 2017;34(9).

43. Toufan M, Pourafkari L, Nasab LG, Esfahani A, Sanaat Z, Nikanfar A, et al. Two-dimensional strain echocardiography for detection of cardiotoxicity in breast cancer patients undergoing chemotherapy. Journal of Cardiovascular and Thoracic Research. 2017;9(1):29-34.

44. Abdel-Rahman O. Patterns of on-treatment cardiac adverse events within three clinical trials of adjuvant anthracycline-based chemotherapy. Breast Cancer. 2018;25(6):723-8.

45. Arciniegas Calle MC, Sandhu NP, Xia H, Cha SS, Pellikka PA, Ye Z, et al. Two-dimensional speckle tracking echocardiography predicts early subclinical cardiotoxicity associated with anthracycline-trastuzumab chemotherapy in patients with breast cancer. BMC Cancer. 2018;18(1).

46. Frères P, Bouznad N, Servais L, Josse C, Wenric S, Poncin A, et al. Variations of circulating cardiac biomarkers during and after anthracycline-containing chemotherapy in breast cancer patients. BMC Cancer. 2018;18(1).

47. Jordan JH, Castellino SM, Meléndez GC, Klepin HD, Ellis LR, Lamar Z, et al. Left ventricular mass change after anthracycline chemotherapy. Circulation: Heart Failure. 2018;11(7).

48. Wittayanukorn S, Qian J, Westrick SC, Billor N, Johnson B, Hansen RA. Prevention of Trastuzumab and Anthracycline-induced Cardiotoxicity Using Angiotensin-converting Enzyme Inhibitors or β-blockers in Older Adults with Breast Cancer. American Journal of Clinical Oncology: Cancer Clinical Trials. 2018;41(9):909-18.

49. Yu LR, Cao Z, Makhoul I, Daniels JR, Klimberg S, Wei JY, et al. Immune response proteins as predictive biomarkers of doxorubicin-induced cardiotoxicity in breast cancer patients. Experimental Biology and Medicine. 2018;243(3):248-55.

50. Abu-Khalaf MM, Safonov A, Stratton J, Wang S, Hatzis C, Park E, et al. Examining the cost-effectiveness of baseline left ventricular function assessment among breast cancer patients undergoing anthracycline-based therapy. Breast Cancer Res Treat. 2019;176(2):261-70.

51. Anber ZNH, Saleh BOM, Al-Rawi SA. The cardiotoxicity effect of different chemotherapeutic regimens in Iraqi patients with breast cancer: A follow up study. Heliyon. 2019;5(8):e02194.

52. Antolín S, Acea B, Albaina L, Concha Á, Santiago P, García-Caballero T, et al. Primary systemic therapy in HER2-positive operable breast cancer using trastuzumab and chemotherapy: Efficacy data, cardiotoxicity and long-term follow-up in 142 patients diagnosed from 2005 to 2016 at a single institution. Breast Cancer: Targets and Therapy. 2019;11:29-42.

53. Canale ML, Camerini A, Huqi A, Lilli A, Bisceglia I, Parrini I, et al. Cardiovascular risk factors and timing of anthracyclines and trastuzumab cardiac toxicity. Anticancer Research. 2019;39(10):5741-5.

54. Clark RA, Marin TS, McCarthy AL, Bradley J, Grover S, Peters R, et al. Cardiotoxicity after cancer treatment: A process map of the patient treatment journey. Cardio-Oncology. 2019;5(1).

55. de Barros MVL, Macedo AVS, Sarvari SI, Faleiros MH, Felipe PT, Silva JLP, et al. Left ventricular regional wall motion abnormality is a strong predictor of cardiotoxicity in breast cancer patients undergoing chemotherapy. Arquivos Brasileiros de Cardiologia. 2019;112(1):50-6.

56. Kim EK, Cho J, Kim JY, Chang SA, Park SJ, Choi JO, et al. Early decline in left ventricular ejection fraction can predict trastuzumab-related cardiotoxicity in patients with breast cancer: A study using 13 years of registry data. Cancer Research and Treatment. 2019;51(2):727-36.

57. Klein R, Nadouri D, Osler E, Johnson C, Dent S, Dwivedi G. Diastolic dysfunction can precede systolic dysfunction on MUGA in cancer patients receiving trastuzumab-based therapy. Nuclear Medicine Communications. 2019;40(1):22-9.

58. Kosalka P, Johnson C, Turek M, Sulpher J, Law A, Botros J, et al. Effect of obesity, dyslipidemia, and diabetes on trastuzumab-related cardiotoxicity in breast cancer. Current Oncology. 2019;26(3):e314-e21.

59. Martinello R, Becco P, Vici P, Airoldi M, Del Mastro L, Garrone O, et al. Trastuzumab-related cardiotoxicity in patients with nonlimiting cardiac comorbidity. Breast Journal. 2019;25(3):444-9.

60. Abdallah IB, Nasr SB, Chourabi C, Boukhris M, Abdallah IB, Zribi A, et al. The predictive value of 2D myocardial strain for epirubicin-induced cardiotoxicity. Journal of Oncology. 2020;2020.

61. Demissei BG, Adusumalli S, Hubbard RA, Denduluri S, Narayan V, Clark AS, et al. Cardiology Involvement in Patients With Breast Cancer Treated With Trastuzumab. JACC: CardioOncology. 2020;2(2):179-89.

62. Eiger D, Pondé NF, Agbor-Tarh D, Moreno-Aspitia A, Piccart M, Hilbers FS, et al. Long-term cardiac outcomes of patients with HER2-positive breast cancer treated in the adjuvant lapatinib and/or trastuzumab Treatment Optimization Trial. British journal of cancer. 2020;122(10):1453‐60.

63. Fang Y, Wang Z, Wu J, Huang O, He J, Zhu L, et al. Factors influencing adjuvant chemotherapy and trastuzumab choice in older human epidermal growth factor receptor 2-positive breast cancer patients. Journal of Cancer. 2020;11(9):2602-9.

64. Foulkes SJ, Howden EJ, Antill Y, Loi S, Salim A, Haykowsky MJ, et al. Exercise as a diagnostic and therapeutic tool for preventing cardiovascular morbidity in breast cancer patients- the BReast cancer EXercise InTervention (BREXIT) trial protocol. BMC cancer. 2020;20(1):655‐.

65. Iqbal M, Victory V, Astutia A, Febrianora M, Karwiky G, Achmad C, et al. Cardiotoxicity by Anthracycline Regimen Chemotherapy Prolonged T Peak to T End Interval. Cardiology Research. 2020;11(5):305-10.

66. Laufer-Perl M, Arnold JH, Mor L, Amrami N, Derakhshesh M, Moshkovits Y, et al. The association of reduced global longitudinal strain with cancer therapy-related cardiac dysfunction among patients receiving cancer therapy. Clinical Research in Cardiology. 2020;109(2):255-62.

67. Sung SY, Lee JH, Yang KH, Seo Y, Kang MY. Coronary event analysis in breast cancer patients who received breast-conserving surgery and post-operative radiotherapy: A Korean nationwide cohort study. Journal of Breast Cancer. 2020;23(3):291-302.

68. Upshaw JN, Hubbard RA, Hu J, Brown JC, Smith AM, Demissei B, et al. Physical activity during and after breast cancer therapy and associations of baseline physical activity with changes in cardiac function by echocardiography. Cancer Medicine. 2020;9(17):6122-31.

69. Yu AF, Flynn JR, Moskowitz CS, Scott JM, Oeffinger KC, Dang CT, et al. Long-term Cardiopulmonary Consequences of Treatment-Induced Cardiotoxicity in Survivors of ERBB2-Positive Breast Cancer. JAMA Cardiology. 2020;5(3):309-17.

70. Yu AF, Moskowitz CS, Lee Chuy K, Yang J, Dang CT, Liu JE, et al. Cardiotoxicity Surveillance and Risk of Heart Failure During HER2 Targeted Therapy. JACC: CardioOncology. 2020;2(2):166-75.

71. Abdel-Qadir H, Tai F, Croxford R, Austin PC, Amir E, Calvillo-Argüelles O, et al. Characteristics and Outcomes of Women Developing Heart Failure After Early Stage Breast Cancer Chemotherapy: A Population-Based Matched Cohort Study. Circ Heart Fail. 2021;14(7):e008110.

72. Araujo-Gutierrez R, Chitturi KR, Xu J, Wang Y, Kinder E, Senapati A, et al. Baseline global longitudinal strain predictive of anthracycline-induced cardiotoxicity. Cardio-Oncology. 2021;7(1).

73. Azhar Y, Agustina H, Hernowo BS. Primary Systemic Therapy for HER2/Neu-Positive Operable Breast Cancer Increases the Number of Breast-Conserving Surgery and Disease-Free Survival: Retrospective Cohort Analysis at Single Institution. Asian Journal of Oncology. 2021;7(2):89-95.

74. de Souza TF, Silva TQ, Antunes-Correa L, Drobni ZD, Costa FO, Dertkigil SSJ, et al. Cardiac magnetic resonance assessment of right ventricular remodeling after anthracycline therapy. Sci Rep. 2021;11(1):17132.

75. Douganiotis G, Grigoriadis S, Kontovinis L, Markopoulou E, Pouptsis A, Papazisis K. Cardiac safety of neoadjuvant chemotherapy with epirubicin and cyclophosphamide followed by docetaxel/pertuzumab/ trastuzumab for HER2-positive breast cancer patients. Journal of BUON. 2021;26(3):714-9.

76. Fradley MG, Alomar M, Kilpatrick MW, Shields B, Tran N, Best A, et al. Patient reported physical and mental health changes associated with a comprehensive cardiovascular risk reduction program for women with breast cancer receiving potentially cardiotoxic chemotherapy. Cardio-oncology. 2021;7(1).

77. Haffadi M, Tawfiq N, Dakir M, Boughafour M, Bouchbika Z, Benchakroun N, et al. Evaluation of cardiac toxicity of systemic treatments in Moroccan patients followed for localized breast cancer: Prospective observational study of 549 cases. The Gulf journal of oncology. 2021;1(35):59-65.

78. Kim DY, Park MS, Youn JC, Lee S, Choi JH, Jung MH, et al. Development and Validation of a Risk Score Model for Predicting the Cardiovascular Outcomes After Breast Cancer Therapy: The CHEMO-RADIAT Score. J Am Heart Assoc. 2021;10(16):e021931.

79. Lee M, Chung WB, Lee JE, Park CS, Park WC, Song BJ, et al. Candesartan and carvedilol for primary prevention of subclinical cardiotoxicity in breast cancer patients without a cardiovascular risk treated with doxorubicin. Cancer medicine. 2021.

80. Puckett LL, Saba SG, Henry S, Rosen S, Rooney E, Filosa SL, et al. Cardiotoxicity screening of long-term, breast cancer survivors-The CAROLE (Cardiac-Related Oncologic Late Effects) Study. Cancer Med. 2021;10(15):5051-61.

81. Reding KW, O’Connell NS, D’Agostino RB, Hundley W, Lucas AR, Ladd AC, et al. Both intermuscular fat and LVEF decline promote heart failure symptoms in cancer survivors. Cardio-Oncology. 2021;7(1).

82. Simões R, Silva LM, de Oliveira AN, Alves MT, Pestana RMC, de Souza IDP, et al. Identification of Clinical and Laboratory Variables Associated with Cardiotoxicity Events Due to Doxorubicin in Breast Cancer Patients: A 1-Year Follow-Up Study. Cardiovasc Toxicol. 2021;21(2):106-14.

83. Subramaniam S, Kong YC, Zaharah H, Uiterwaal C, Richard A, Taib NA, et al. Baseline cardiovascular comorbidities, and the influence on cancer treatment decision-making in women with breast cancer. Ecancermedicalscience. 2021;15:1293.

84. Sulaiman L, Hesham D, Abdel Hamid M, Youssef G. The combined role of NT-proBNP and LV-GLS in the detection of early subtle chemotherapy-induced cardiotoxicity in breast cancer female patients. Egyptian Heart Journal. 2021;73(1).

85. Tang M, Schaffer AL, Kiely BE, Daniels B, Lee CK, Simes RJ, et al. Cardiac assessment in Australian patients receiving (neo)adjuvant trastuzumab for HER2-positive early breast cancer: a population-based study. Breast Cancer Research and Treatment. 2021;187(3):893-902.

86. Vaitiekus D, Muckiene G, Vaitiekiene A, Sereikaite L, Inciuraite R, Insodaite R, et al. HFE Gene Variants' Impact on Anthracycline-Based Chemotherapy-Induced Subclinical Cardiotoxicity. Cardiovascular Toxicology. 2021;21(1):59-66.

87. Varghese SS, Johnston WJ, Eekhoudt CR, Keats MR, Jassal DS, Grandy SA. Exercise to Reduce Anthracycline-Mediated Cardiovascular Complications in Breast Cancer Survivors. Curr Oncol. 2021;28(5):4139-56.

88. Vo JB, Kenzik KM, Landier W, Raju D, Kirklin JK, Meneses K. Excess heart age in young breast cancer survivors over 2-year follow-up. Cancer Causes Control. 2021;32(6):617-26.

89. Yao RJR, Gibson J, Simmons C, Davis MK. Management strategies and clinical outcomes in breast cancer patients who develop left ventricular dysfunction during trastuzumab therapy. Cardio-Oncology. 2021;7(1).

90. Yoodee J, Sookprasert A, Sanguanboonyaphong P, Chanthawong S, Seateaw M, Subongkot S. An exploration of heart failure risk in breast cancer patients receiving anthracyclines with or without trastuzumab in thailand: A retrospective study. Clinics and Practice. 2021;11(3):484-93.
